# Mitochondrial dysfunction-related metabolite methylmalonic acid is associated with decreased cognitive performance

**DOI:** 10.1371/journal.pone.0332987

**Published:** 2025-10-17

**Authors:** Yige Liu, Jiaxin Wang, Pengyan Wu, Fan Tang, Wanlu Guo, Ruishan Li, Xinran Wang, Yiying Zhang, Shaohong Fang

**Affiliations:** 1 Department of Cardiology, Second Affiliated Hospital of Harbin Medical University, Harbin, Heilongjiang, China; 2 The Key Laboratory of Myocardial Ischemia, Chinese Ministry of Education, Harbin, Heilongjiang, China; 3 Department of Epidemiology and Biostatistics, School of Public Health, Jiamusi University, Jiamusi, Heilongjiang, China; Fudan University, CHINA

## Abstract

**Purpose:**

Methylmalonic acid (MMA), a mitochondrial metabolite derived from propionate metabolism, has been implicated in fatal neurodegeneration in children with congenital methylmalonic acidemia. However, its clinical relevance in chronic cognitive decline remains poorly understood. This study aims to investigate the association between MMA levels, its metabolic coenzyme vitamin B12, and cognitive impairment in an elderly population.

**Patients and methods:**

The cohort comprised 2,762 participants aged 60 and older, who underwent assessments using the Digit Symbol Substitution Test (DSST), Animal Fluency test (AFT), Consortium to Establish a Registry for Alzheimer’s disease (CERAD) Immediate Recall (CERAD-IR), CERAD Delayed Recall (CERAD-DR), with low cognitive performance defined as the bottom quartile. Generalized linear regression models were applied to explore potential associations. Additionally, differentially expressed genes (DEGs) in MMA-related propionate metabolic process were identified from the RNA expression profiles in frontal cortex from 56 Alzheimer’s disease and 44 non-dementia controls.

**Results:**

Among the 2,762 participants, with a mean age of 69.2 years and 53.8% female, elevated MMA levels were significantly associated with an increased likelihood of low cognitive performance across multiple tests: DSST (OR = 1.95; 95% CI: 1.41–2.73), AFT (OR = 1.51; 95% CI: 1.01–2.25), CERAD-IR (OR = 1.58; 95% CI: 1.19–2.09), and CERAD-DR (OR = 1.56; 95% CI: 1.12–2.18). High serum vitamin B12 levels were associated with an increased likelihood of impaired CERAD-IR scores, although dietary B12 supplementation did not correlate with cognitive performance. Stratified analyses revealed a stronger relationship between MMA and cognitive decline in males, individuals over 75 years, and those with elevated serum and dietary vitamin B12 levels. DEG analysis identified genes related to MMA synthesis and mitochondrial function in cognitively impaired individuals, with significant enrichment in mitochondrial propionate metabolism pathways.

**Conclusion:**

The findings indicate that elevated MMA levels are linked to cognitive decline in older adults, potentially through mitochondrial dysfunction. These results underscore the clinical importance of MMA in the context of chronic cognitive impairment.

## Introduction

As global aging accelerates, senile dementia has become a major public health challenge for the elderly population. According to the World Alzheimer Report, the global prevalence of dementia is expected to reach approximately 130 million by 2050, doubling every two decades [1]. Currently, therapeutic options for Alzheimer’s disease (AD) fail to slow or prevent the fatal neuronal damage that underpins AD symptoms [2]. Cognitive impairment, from mild to severe, describes a decline in abilities related to memory, learning, attention, and decision-making [3,4]. Early detection and intervention in cognitive decline could substantially reduce healthcare costs and improve the quality of life for elderly individuals. Recent studies have increasingly highlighted the critical role of metabolic alterations in the pathogenesis and progression of AD and related cognitive impairments [5,6]. This study aims to identify potential biomarkers associated with cognitive impairment, providing a scientific basis for its prevention and delay.

Methylmalonic acid (MMA), a toxic metabolite linked to mitochondrial dysfunction, disrupts the involvement of succinyl-CoA in the tricarboxylic acid (TCA) cycle, impairing mitochondrial energy metabolism. The accumulation of MMA is associated with widespread systemic damage, particularly affecting the central nervous system [7]. In a study of 43 individuals with congenital MMAemia, cognitive impairment was significantly correlated with elevated MMA levels, as assessed using the Wechsler Intelligence Scale. Further research has demonstrated that in pediatric congenital MMAemia, elevated MMA not only disrupts mitochondrial energy production in the nervous system but also increases intracellular reactive oxygen species [8], (ROS), leading to neuronal apoptosis and cognitive decline. Similar morphological changes in cortical neurons, along with increased apoptosis, were observed in rat models with elevated MMA levels [9,10]. Transcriptomic analysis of these models revealed significant enrichment in apoptosis-related pathways, including the mitogen-activated protein kinase (MAPK) pathway and the P53 signaling pathway, highlighting the neurotoxic mechanisms through which MMA induces cognitive deficits [11]. However, most studies on MMA-induced neuronal damage focus primarily on pediatric populations, leaving the relationship between MMA and chronic cognitive impairment in adults largely unexplored, which motivates the present investigation [12–15]. MMA also serves as a biomarker for vitamin B12 deficiency, which, in conjunction with methylmalonyl-CoA mutase (MMUT), catalyzes the conversion of MMA to succinyl-CoA for mitochondrial energy production [16]. A well-established link exists between vitamin B12 deficiency and cognitive decline, particularly among subsets of the elderly with poor responsiveness to B12 [17]. Some researchers suggest that vitamin B12 supplementation may alleviate cognitive impairment by lowering MMA levels and improving neuronal energy metabolism [18]. However, numerous clinical trials have shown that vitamin B12 supplementation does not improve neurological function, with the controversy often attributed to a focus on serum vitamin B12 levels while overlooking the effects of dietary B12 and supplements on cognition [19–21]. Therefore, this study aims to comprehensively assess the cross-sectional relationship between MMA levels, serum and dietary vitamin B12 intake, dietary B12 supplementation, and cognitive decline in older adults.

## Materials and methods

### Study population

A cross-sectional study was conducted using data from the National Health and Nutrition Examination Survey (NHANES) for the years 2011–2012 and 2013–2014. This comprehensive biennial survey, conducted in the United States, provides a representative sample of non-institutionalized civilians. All data collection procedures were approved by the research ethics review board at the National Center for Health Statistics, and written informed consent was obtained from each participant [22].

**Fig 1:**
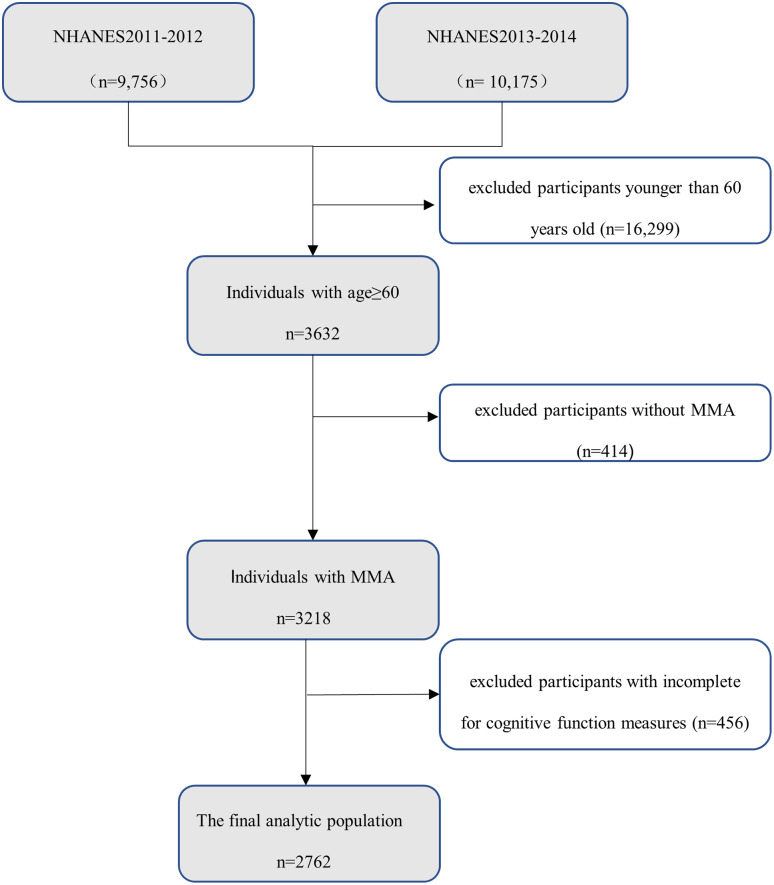
Flow of study.

Data from two survey cycles (2011–2012 and 2013–2014) that included information on MMA and cognitive function were combined for analysis. A total of 3,632 individuals aged 60 and older participated in the study. Participants lacking MMA measurement data (n = 414) or with incomplete or unreliable cognitive test results (n = 456) were excluded. The final analysis was based on 2,762 adults aged ≥ 60 years (1,355 males and 1,407 females) (Fig 1).

### MMA and vitamin B12-related biomarkers

Blood samples were collected under standardized protocols via venipuncture at the mobile examination center (MEC). MMA levels were analyzed by mass spectrometry in both plasma and/or serum, with preference given to plasma. Previous research has shown that MMA concentrations in both serum and plasma share similar reference ranges and coefficients of variation [23].

Blood samples were collected from adults aged 20 years and older, then processed, stored, and shipped to the Division of Laboratory Sciences at the National Center for Environmental Health, Centers for Disease Control and Prevention, in Atlanta for analysis. Samples were stored under proper conditions at −30°C until they were transferred to the National Center for Environmental Health. Serum vitamin B12 levels were measured using the Elecsys Vitamin B12 assay, a competitive electrochemiluminescence immunoassay designed for the specific detection of vitamin B12 and intrinsic factors [24]. Laboratory personnel, including certified medical technologists and phlebotomists, received extensive training prior to their employment at the MEC.

Dietary B12 intake was assessed using a 24-hour dietary recall interview conducted by NHANES through a computer-based system [25]. This assessment aimed to capture comprehensive data on the consumption of all foods, beverages, and supplements within the 24-hour period prior to the interview, from midnight to midnight. The interview followed a multipack format, with all interviewers holding at least a college degree and formal training in food and nutrition.

To determine dietary supplement use, participants were queried about their supplement consumption over the past 30 days. Data on food intake during the 24-hour recall period were collected during an in-person session at the MEC (day 1) and followed up with telephone interviews conducted 3–10 days later (day 2) [24]. Supplement usage data were utilized to identify the types and quantities of dietary supplements consumed, as well as to estimate nutrient intake from these supplements. Participants who reported using supplements were asked to provide details, including the supplement name, frequency of use, and usual dosage. Specifically, information on vitamin B12 supplement use was extracted for analysis.

### Cognitive performance assessment

In this study, cognitive function assessment was limited to individuals aged 60 years and above. The evaluation included the Word Learning and Recall modules from the Consortium to Establish a Registry for Alzheimer’s Disease (CERAD-WL), along with the animal fluency test (AFT) and the digit symbol substitution test (DSST).

The CERAD-WL assesses both immediate and delayed learning abilities following the presentation of new verbal information [26]. This assessment comprises three consecutive immediate recall learning tests (CERAD-IR) and a delayed word recall test (CERAD-DR). During the CERAD-IR learning tests, participants were asked to verbally repeat a list of ten unrelated words. After the presentation of the words, participants were prompted to recall as many words as possible. The total number of recalled words determined their score for each trial, which could range from 0 to 10. Thus, the overall CERAD-IR score could range between 0 and 30. The CERAD-DR was administered approximately 8–10 minutes after the word learning trials, following the completion of the other two cognitive tasks (AFT and DSST). This delayed recall test has a score range of 0–10 [27].

The AFT evaluates categorical verbal fluency [28]. Participants were instructed to name as many animals as they could within a 60-second period, with the total number of animals named determining the participant’s AFT score.

The DSST, a performance module from the Wechsler Adult Intelligence Scale (WAIS-III), evaluates processing speed, sustained attention, and working memory [29]. The task was presented in a paper-based format, with a key at the top that paired nine numbers with specific symbols. Participants were given 120 seconds to replicate the corresponding symbols in the 133 adjacent boxes associated with the numbers. The DSST score was based on the total number of correct symbol-number matches.

Currently, there is no universally accepted standard for defining cutoff points for the CERAD, AFT, and DSST tests to identify low cognitive performance. Therefore, the 25^th^ percentile of the scores were applied as the cutoff, reflecting the lowest quartile, in accordance with methods used in previous studies [30]. For each cognitive assessment, participants were divided into two groups: those in the low cognitive performance group, who scored below the 25^th^ percentile, and those in the normal cognitive performance group, who scored above the 25^th^ percentile.

### Covariates

Additional variables recorded included age, sex (male, female), race/ethnicity (Mexican-American, other Hispanic, non-Hispanic white, non-Hispanic black), education level (less than high school, high school graduate, more than high school), body mass index (BMI), smoking status (never, former, current), alcohol consumption (male ≥ 20 g/day, female ≥ 10 g/day), Sufficient physical activity (at least 150 minutes moderate-to-vigorous physical activity per week as current guideline recommended), systolic blood pressure, the ratio of high-density lipoprotein (HDL) to total serum cholesterol, glomerular filtration rate (GFR), type 2 diabetes, coronary heart disease (CHD), and stroke. A history of type 2 diabetes, stroke, or CHD was defined as a self-reported diagnosis confirmed by a healthcare provider.

### Statistical methods

Statistical analyses were conducted using STATA software (version 15.19), with a significance threshold set at 5%. To facilitate the generalization of findings to the broader noninstitutionalized U.S. population, complex sample analyses were performed. Continuous variables were expressed as means with standard errors (SE), while categorical variables were reported as proportions. The dose-response relationship between MMA and cognitive function was elucidated by utilizing restricted cubic spline (RCS). Both crude and adjusted estimates (odds ratios [OR] or regression coefficients with 95% confidence intervals [CI]) were calculated through generalized linear regression models, assessing the associations between MMA levels and cognitive function, with low cognitive performance defined as scoring below the lowest weighted quartile, and cognitive function assessed as continuous variables. Four multivariable models adjusted for conventional risk factors were employed. Model 1 adjusted for demographic factors (age, sex, and race/ethnicity); Model 2 further adjusted for education, smoking, alcohol consumption, physical activity, BMI, and systolic blood pressure; Model 3 included additional adjustments for type 2 diabetes, stroke, and CHD; and Model 4 additionally controlled for serum vitamin B12 concentrations, the cholesterol-to-HDL ratio, and glomerular filtration rate.

According to the previously reported cutoff values: normal range of B12(≥ 250 pmol/L), high range of B12(> 800 pmol/L) [31]. Generalized linear regression models were applied to investigate the associations between serum cobalamin (both continuous and by tertiles), cobalamin intake from foods (by tertiles), cobalamin supplements (use vs. nonuse), and cognitive function. To investigate potential effect modification, a priori subgroup analyses were conducted by age, sex, dietary and serum B12 levels, supplement use, and estimated glomerular filtration rate (eGFR), with adjustments for relevant variables. Sensitivity analysis of participants with an eGFR < 15 mL/min/1.73 m2, indicative of kidney failure requiring dialysis, was performed. Finally, mediation analysis was conducted within the crude model to explore the role of eGFR as a mediator in explaining the statistical significance of the logistic regression results.

### Data acquisition differential gene expression analysis (DEGs) and enrichment analysis

The series matrix and annotation files were retrieved from the GEO public database of NCBI. The dataset GSE122063, generated by the GPL16699 platform, includes RNA expression data from frontal cortex samples collected from individuals who died with or without Alzheimer’s disease (56 Alzheimer’s disease and 44 non-dementia (ND) controls). The data underwent background correction, array normalization, and log2 transformation. Differentially expressed genes (DEGs) were identified using the “limma” R package within R software. Principal component analysis (PCA) was performed with the “factoextra” R package. To balance statistical rigor with exploratory breadth, we applied p < 0.05 and |log2 (fold-change) | > 1 as thresholds for DEG selection. The “ggplot2” package was employed for DEG visualization, generating a volcano plot. Biological processes (BPs) of the Gene Ontology (GO) and Kyoto Encyclopedia of Genes and Genomes (KEGG) pathways were enriched using the “clusterProfiler” package, with results visualized via the “enrichplot” package.

## Results

### Participant characteristics in NHANES

This study enrolled 2,762 participants aged 60 years and older, with baseline characteristics of circulating MMA detailed in Table 1. The cohort consisted of 53.81% females and 46.19% males. A history of CHD, type 2 diabetes, and stroke was reported in 15.05%, 21.02%, and 0.91% of participants, respectively. The analysis showed that Age, Race/ethnicity, Education, Physical activity, Systolic BP, TC/HDL-C ratio, serum vitamin B12, B12 supplement use, estimated glomerular filtration rate (eGFR, mL/min per 1.73m2), CHD, and stroke indicators were significantly associated with circulating methylmalonic acid levels. Furthermore, BMI (P = 0.4), smoking status (P = 0.9), alcohol consumption (P = 1.0), B12 food intake (P = 0.6), and type 2 diabetes (P = 0.6) showed no significant correlation with MMA levels.

**Table 1:** Baseline characteristics of participants in NHANES 2011-2014 by baseline methylmalonic acid levels.

	Circulating methylmalonic acid (MMA, nmol/L)				
Variables	Q1 < 128 (n = 705)	128 ≤ Q2 < 166(nmol/L) (n = 681)	166 ≤ Q3 < 229(nmol/L) (n = 686)	Q4 ≥ 229(nmol/L) (n = 690)	*p* for trend
Age, y	66.8 ± 0.3	68.3 ± 0.3	69.9 ± 0.5	71.5 ± 0.3	<0.001
Sex, %					
Female	55.3	53.3	52.2	54.8	
Male	44.7	46.7	47.9	45.2	0.8
Race/ethnicity, %					
Non-Hispanic White	70.4	78.5	85.6	85.4	<0.001
Non-Hispanic Black	11.1	9.8	5.6	5.6	<0.001
Hispanic-Mexican	5.0	3.3	2.8	2.6	<0.001
Other Ethnicity	13.5	8.5	6.1	6.4	<0.001
Education, %					<0.001
Less than high school	13.2	14.3	15.5	19.6	
High school	19.8	20.6	23.0	25.6	
More than high school	67.0	65.1	61.6	54.8	
BMI, kg/m^2^	29.2 ± 0.4	28.5 ± 0.3	29.1 ± 0.3	29.4 ± 0.3	0.4
Smoking status					0.9
Never	50.6	48.2	49.9	51.0	
Former	40.4	38.4	38.5	38.4	
Current	9.0	13.4	11.6	10.6	
Alcohol consumption, g/d	6.6	7.9	5.9	7.2	1.0
Sufficient physical activity, %	33.1	32.2	31.1	22.8	<0.05
Systolic BP, mmHg	129.9 ± 0.9	129.9 ± 1.0	131.4 ± 1.4	133.3 ± 1.0	<0.05
TC/HDL-C ratio	1.3 ± 0.1	1.4 ± 0.1	1.6 ± 0.1	1.6 ± 0.1	<0.05
Serum vitamin B12, pmol/L	647.7 ± 40.9	568.0 ± 28.9	456.2 ± 10.6	402.1 ± 20.9	<0.001
B12 intake from foods(μg/day)	4.8 ± 0.4	5.0 ± 0.2	4.8 ± 0.2	5.7 ± 1.4	0.6
B12 supplements, %	53.1	53.9	41.2	35.5	<0.001
eGFR, mL/min per 1.73m²	82.9 ± 0.1	76.7 ± 0.6	71.8 ± 0.7	62.7 ± 1.0	<0.001
Type 2 Diabetes, %	25.1	18.6	17.2	24.0	0.6
CHD, %	10.5	13.8	16.3	19.2	<0.001
Stroked, %	0.2	1.0	0.5	1.9	<0.05

Variables are presented as the weighted proportion (%) or mean ± SE.

P for trend was estimated with linear regression for continuous variables and with logistic regression for categorical variables.

Abbreviations: BMI, body mass index; BP, blood pressure; TC/HDL-C ratio, the ratio of high-density lipoprotein serum total cholesterol to high-density lipoprotein; eGFR, estimated glomerular filtration rate; CHD, coronary heart disease.

### Relationship between methylmalonic acid, vitamin B12 and cognitive impairment

**Fig 2:**
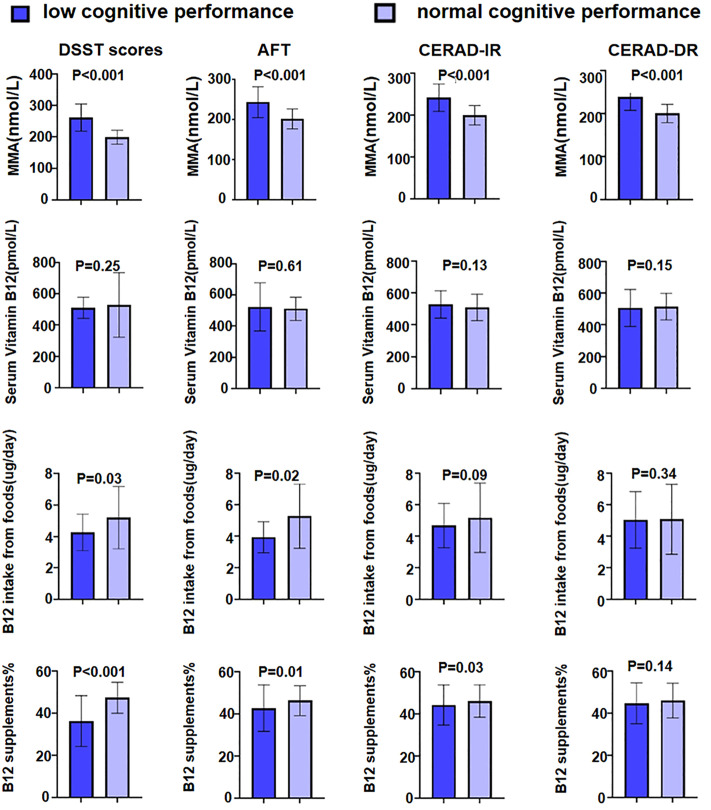
Elevated concentrations of serum MMA among participants with decreased cognitive performance. Error bars are 95%CIs. The relationship between cognitive scores and MMA in the mean standard deviation.

The weighted means or proportions of serum MMA, serum vitamin B12, dietary B12 intake, and B12 supplementation in adults with or without low cognitive performance are presented in Fig 2. Participants with low cognitive performance across all cognitive dimensions exhibited elevated serum MMA concentrations. However, no significant correlation was observed between serum vitamin B12 levels, dietary B12 intake, or B12 supplementation and the likelihood of low cognitive performance (Fig 2). To investigate the dose-response relationship between MMA and cognitive function, RCS was first performed and its number of knots was set at 3 (S1 Fig). Higher levels of serum MMA were associated with cognitive function. We next investigated the associations between MMA levels and low cognitive performance (Table 2). In both crude analyses and after adjusting for demographic variables (age, sex, race/ethnicity), elevated circulating MMA levels were significantly associated with increased odds of poor performance on DSST scores, AFT, CERAD-IR, and CERAD-DR.

**Table 2:** The Relationship Between Methylmalonic Acid and Cognitions in NHANES 2011–2014.

	Circulating methylmalonic acid (nmol/L)				
	Q1	Q2	Q3	Q4	P for tred
	OR (95%CI)	OR (95%CI)	OR (95%CI)	OR (95%CI)	
DSST scores					
Crude	1.00(Ref.)	0.80 (0.58 to 1.12)	1.05 (0.74 to 1.49)	2.08 (1.61 to 2.68)	<0.001
Model 1	1.00(Ref.)	0.76 (0.53 to 1.11)	1.03 (0.69 to 1.53)	1.95 (1.41 to 2.73)	<0.001
Model 2	1.00(Ref.)	0.78 (0.49 to 1.24)	0.93 (0.6 to 1.42)	1.45 (0.93 to 2.25)	0.015
Model 3	1.00(Ref.)	0.83 (0.51 to 1.31)	1.00 (0.66 to 1.50)	1.60 (1.00 to 2.54)	0.01
AFT					
Crude	1.00(Ref.)	0.97 (0.62 to 1.53)	1.10 (0.75 to 1.61)	1.78 (1.22 to 2.61)	<0.001
Model 1	1.00(Ref.)	0.94 (0.59 to 1.51)	1.03 (0.69 to 1.52)	1.51 (1.01 to 2.25)^*^	0.001
Model 2	1.00(Ref.)	0.91 (0.56 to 1.46)	0.89 (0.61 to 1.3)	1.13 (0.74 to 1.71)	0.187
Model 3	1.00(Ref.)	0.91 (0.56 to 1.47)	0.90 (0.62 to 1.30)	1.15 (0.78 to 1.68)	0.114
CERAD: score immediate recall					
Crude	1.00(Ref.)	1.10 (0.76 to 1.61)	1.49 (1.05 to 2.10)	2.16 (1.70 to 2.75)	<0.001
Model 1	1.00(Ref.)	0.99 (0.65 to 1.51)	1.21 (0.85 to 1.71)	1.58 (1.19 to 2.09)	0.002
Model 2	1.00(Ref.)	0.97 (0.62 to 1.53)	1.03 (0.71 to 1.50)	1.20 (0.86 to 1.67)	0.111
Model 3	1.00(Ref.)	0.98 (0.62 to 1.55)	1.06 (0.72 to 1.55)	1.24 (0.89 to 1.73)	0.091
CERAD: score delayed recall					
Crude	1.00(Ref.)	1.27 (0.81 to 1.98)	1.76 (1.20 to 2.58)	2.33 (1.76 to 3.08)	0.001
Model 1	1.00(Ref.)	1.10 (0.69 to 1.75)	1.34 (0.90 to 1.99)	1.56 (1.12 to 2.18)	0.091
Model 2	1.00(Ref.)	1.14 (0.7 to 1.85)	1.24 (0.77 to 2.01)	1.33 (0.89 to 1.99)	0.487
Model 3	1.00(Ref.)	1.14 (0.70 to 1.87)	1.24 (0.76 to 2.01)	1.30 (0.88 to 1.92)	0.413

Calculated using binary logistic regression;

Ref, treating the bottom group (the lowest quartile of MMA) as the reference;

Abbreviations: CI, confidence interval; OR, odds ratio; DSST, Digit Symbol Substitution Test; AFT, Animal Fluency test; CERAD, Consortium to Establish a Registry for Alzheimer’s Disease;

Model 1, adjusted for age (years, continuous), sex (female or male), and race/ethnicity (non-Hispanic white, black, Hispanic-Mexican, or other).

Model 2, additionally adjusted for education level (less than high school, high school graduate, more than high school), smoking status (never, former, current), meeting recommended volume of physical activity (no/yes), alcohol consumption (male ≥ 20g/day, and female ≥ 10g/day), body mass index (kg/m2, continuous), systolic blood pressure (mmHg, continuous), the ratio of high-density lipoprotein to total cholesterol (ratio, continuous), type 2 diabetes (no/yes), stroked (no/yes), estimated glomerular filtration rate (≥ 60mL/min/1.73m², and <60 mL/min/1.73m²).

Model 3, additionally adjusted for serum vitamin B12 (pmol/L, continuous).

*P < 0.05, **P < 0.001

After further adjusting for all confounders, there still was a significant correlation between higher MMA levels and poorer DSST performance (S1 Table). High MMA concentrations were notably associated with decreased decline cognition performance as assessed by DSST scores (Q4 vs. Q1 OR: 1.62; 95% CI: 1.07–2.45) and CERAD-IR scores (Q4 vs. Q1 OR: 1.37; 95% CI: 1.01–1.86), suggesting that the link between MMA and low cognitive performance is independent of serum vitamin B12 levels. Further analysis of the effects of various forms of vitamin B12 on cognitive function revealed that adults with serum vitamin B12 levels above 800 pmol/L were only significantly more likely to experience cognitive impairment in CERAD-IR, compared to those with serum B12 levels below 250 pmol/L. Dietary vitamin B12 intake was associated with a reduced likelihood of low cognitive performance in AFT. However, B12 supplementation did not correlate with a decrease in cognitive performance (S2-S4 Tables), indicating a potentially non-linear relationship between vitamin B12 levels and cognitive function, which may be attributable to the modulatory effect of age on cobalamin absorption efficiency.

The B12-related stratified analyses were conducted to assess the relationship between MMA and cognition (S5-S7 Tables). In individuals with serum vitamin B12 levels exceeding 400 pmol/L, MMA was significantly associated with reduced performance on DSST scores. Among individuals with dietary B12 intake ≥ 5.07 μg/d, the highest quartile of MMA was significantly linked to lower cognition performance as assessed by DSST scores (Q4 vs. Q1 OR: 2.91, 95% CI: 1.55–5.54), AFT scores (Q4 vs. Q1 OR: 2.34, 95% CI: 1.19–4.58), CERAD-IR scores (Q4 vs. Q1 OR: 2.64; 95% CI: 1.63–4.30), and CERAD-DR scores (Q4 vs. Q1 OR: 2.42; 95% CI: 1.35–4.35). These results suggest a significant relationship between MMA elevation and cognitive impairment among individuals with serum vitamin B12 > 400 pmol/L, no use of dietary supplements, and dietary vitamin B12 intake ≥ 5.07 μg/d.

The gender-related subgroup analysis of MMA and cognition is shown in S8 Table [32]. The highest quartile of MMA was significantly associated with decreased performance on DSST scores (Q4 vs. Q1 OR: 2.74, 95% CI: 1.56–4.81), CERAD-IR scores (Q4 vs. Q1 OR: 1.87, 95% CI: 1.15–3.04), and CERAD-DR scores (Q4 vs. Q1 OR: 2.06, 95% CI: 1.19–3.58), with this association being significant only in males (S8 Table). Among individuals older than 75 years, the highest quartile of MMA was significantly associated with lower cognitive performance on DSST, CERAD-IR, and CERAD-DR scores (S9 Table).

### Mitochondrial dysfunction and abnormal MMA-related metabolism in autopsied brain tissues from patients with Alzheimer’s disease

**Fig 3:**
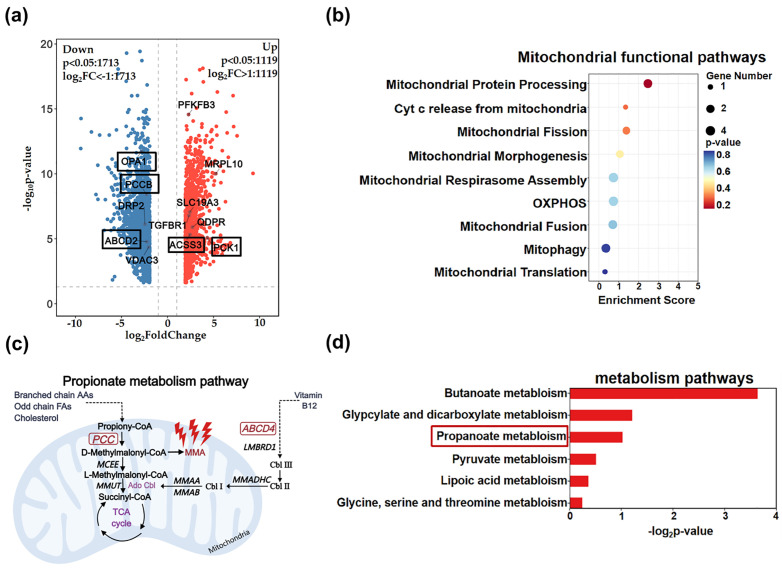
Differential gene expression and enrichment analysis in the RNA expression profiles of frontal cortex from patients with or without Alzheimer’s disease. (a) Volcano plot of DEGs between AD and ND groups in GSE122063. (b) GO enrichment analysis of mitochondrial function in Alzheimer’s disease patients. (c) Propionic acid metabolism pattern diagram; (d) Metabolic KEGG Enrichment Analysis of Alzheimer’s Disease Patients.

RNA sequence profiling was analyzed in autopsied brain tissues of frontal and temporal cortex from 56 Alzheimer’s disease and 44 non-dementia (ND) controls (GSE122063). Among the detectable 18,378 genes, we identified 2,832 DEGs, comprising 1,119 significantly upregulated genes and 1,713 downregulated genes (p-values < 0.05 and log2 fold changes (Log2FC) > 1, Fig 3a). Special attention was given to genes associated with MMA-related propionate metabolism, such as PCC, ABCD, and TGFBR1, as well as those closely linked to mitochondrial function, including OPA1, VDAC3, BRP2, and MRPL10, which were annotated accordingly. To explore the biological functions and potential signaling pathways of these DEGs, we further conducted GO and KEGG analyses. The GO analysis revealed significant enrichment of DEGs in several biological processes related to mitochondrial function, including mitochondrial protein processing, cytochrome C release from mitochondria, mitochondrial fission and morphogenesis, mitochondrial respirasome assembly, ATP synthesis coupled with electron transport, mitochondrial fusion, mitochondrial autophagy, and mitochondrial translation (Fig 3b). These results suggest that mitochondrial dysfunction may play a critical role in cognitive impairment. MMA, as a by-product of the propionate metabolic pathway, is generated when branched-chain amino acids, fatty acids, and cholesterol are catabolized in the mitochondria to form propionyl-CoA (Fig 3c), which is then converted to d-methylmalonyl-CoA by propionyl-CoA carboxylase (PCC). The conversion of d-methylmalonyl-CoA to its L-isomer is mediated by methylmalonyl-CoA exonuclease (MCEE). Methylmalonyl-CoA is further transformed into succinyl-CoA by methylmalonyl-CoA mutase (MMUT), and subsequently entering the tricarboxylic acid cycle. Vitamin B12 is converted into adenosyl cobalamin, a cofactor essential for Mmut activity. KEGG pathway enrichment analysis in patients with AD revealed significant impairment in the propionate metabolic pathway (Fig 3d). Additionally, differential expression of PCC, a pivotal gene in the propionate metabolic pathway, and ABCD4, a gene involved in vitamin B12 processing, was reduced in the brain tissues of AD patients (Fig 3a). These results suggest that the accumulation of MMA, driven by disrupted propionate metabolism may significantly contribute to mitochondrial dysfunction and cognitive impairment.

### Mediation analyses of eGFR for the relationship between methylmalonic acid and cognitions

**Fig 4:**
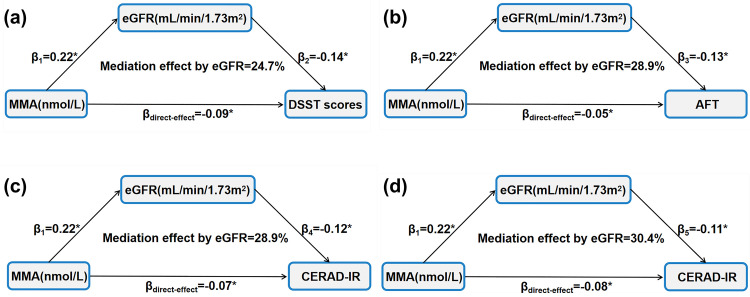
The Mediation analysis for the Relationship between Methylmalonic Acid and Cognitions via eGFR. Calculated using sgmediation2. Data were standardized regression coefficients; (a)The mediation analysis for the association between MMA and DSST. (b)The mediation analysis for the association between MMA and AFT. (c)The mediation analysis for the association between MMA and CERAD-IR. (b)The mediation analysis for the association between MA and CERAD-DR. The association with cognitive function was estimated with weighted Logistic regression analysis, adjusted for age (years, continuous), sex (female or male), race/ethnicity (non-Hispanic white, black, Hispanic-Mexican, or other), education level (less than high school, high school graduate, more than high school), smoking status (never, former, current), physical activity (at least 150 minutes of exercise per week), alcohol consumption (male ≥ 20g/day, female ≥ 10g/day), body mass index (kg/m2, continuous), systolic blood pressure (mmHg, continuous), coronary heart disease (no/yes), stroked (no/yes), type 2 diabetes (no/yes), serum vitamin B12 (pmol/L, continuous), the ratio of high-density lipoprotein to total cholesterol, estimated glomerular filtration rate (≥ 60mL/min/1.73m², < 60 mL/min/1.73m²). Arrows indicate the effect pathway. *P < 0.05.

The kidney function plays a pivotal role in eliminating toxins from the body and promoting cerebral circulation. Research has established a negative correlation between eGFR and cognitive function, particularly in middle-aged and elderly populations [33,34]. Additionally, a negative relationship has been demonstrated between eGFR and cerebrospinal fluid biomarkers associated with AD, such as Aβ42, t-tau, p-tau, t-tau/Aβ42, and p-tau/Aβ42 [35]. These results suggest that renal dysfunction may accelerate cognitive decline. Previous studies reported that MMA has detrimental effects on kidney function, including causing tubulointerstitial nephritis [36]. Given the established renal impact of MMA, the mediation of eGFR on the relationship between MMA and cognition was explored (Fig 4). The total indirect effect of eGFR was calculated with β1 to β5, revealing that eGFR-mediated effects accounted for 24.7%, 28.9%, 28.9%, and 30.4% of the total effects of MMA on DSST scores, AFT, CERAD-IR, and CERAD-DR, respectively. These results indicate that eGFR partly mediates the relationship between MMA accumulation and cognitive decline.

## Discussion

This study yielded several key findings: 1. A significant association exists between elevated levels of blood MMA and the increased odds of cognitive decline. This phenomenon can be attributed to the established neurotoxic effects of MMA in genetic metabolic diseases. 2. Stratified analysis revealed that the relationship between low cognitive ability and MMA was more pronounced in individuals with higher serum vitamin B12 levels and higher dietary B12 intake. These results suggest that elevated MMA levels contribute to an increased risk of cognitive decline in older adults, likely due to impaired vitamin B12 responsiveness rather than a deficiency in the vitamin itself. 3. Gene-level analysis identified differences in the expression of genes related to MMA metabolism, as well as mitochondrial function, in individuals with AD. This study provides a novel clinical perspective on the uncertain effects of MMA on cognitive decline in older adults and possible mechanisms underlying this link.

Mitochondrial dysfunction is a proposed mechanism linking MMA to cognitive impairment. Despite comprising only about 2% of the body’s total weight, the brain consumes roughly 20% of the body’s energy [37]. Mitochondria, as the central hub for cellular energy metabolism, play a critical role in sustaining cellular functions. In a resting state, cortical neurons exhibit particularly high energy demands, consuming approximately 4.7 x 109 ATP molecules per second [38]. This underscores the reliance of the nervous system on mitochondrial function. Consequently, any disturbance in mitochondrial function may have detrimental effects on high-energy-demand cell types, potentially leading to a variety of both inherited and acquired diseases [39]. MMA is recognized as a marker of mitochondrial damage and oxidative stress. The “mitochondrial disorder” theory of congenital methylmalonic acidemia places mitochondrial dysfunction and oxidative stress at the forefront of the disease mechanism. This supports the idea that investigating MMA production pathways and inhibiting oxidative stress could help mitigate symptoms of cognitive impairment. In a mouse model of ischemia-reperfusion (I/R) injury, MMA exacerbated myocardial oxidative stress and ferroptosis by activating the NADPH oxidase 2/4 (NOX2/4) pathway, leading to increased myocardial infarct size and cardiac dysfunction. To address this pathological mechanism, various antioxidant therapeutic strategies have been explored to reduce ROS-induced neurological damage. For example, the Pt@PCN222-Mn nanozyme, functioning as a cascade catalyst, effectively scavenges ROS, alleviating neurologic ischemia-reperfusion injury. The PdZn/CoSA-NC nanozyme enhances free radical adsorption, lowers reaction energy, and accelerates the catalytic activities of superoxide dismutase (SOD), catalase (CAT), and glutathione peroxidase (GPx) by optimizing electron transfer and the exposure of active sites, demonstrating superior efficacy compared to traditional catalysts. Furthermore, dietary vitamin supplementation offers a potential therapeutic approach to mitigate neurologic injury. Vitamin B12 and levocarnitine, as dietary supplements, protect neuronal structure and enhance energy metabolism, thus reducing neuronal damage. In summary, inhibiting ROS production represents an effective strategy to alleviate cognitive impairments induced by MMA, and dietary vitamin supplementation offers a promising therapeutic avenue for mitigating neurologic injury.

Beyond Alzheimer’s disease, elevated MMA levels have also been implicated in other neurodegenerative disorders, most notably Parkinson’s disease (PD). Clinical studies have reported higher circulating MMA concentrations in patients with PD compared to healthy controls, and these elevations correlated with more severe motor dysfunction and faster disease progression [40,41]. Mechanistically, MMA has been shown to exacerbate mitochondrial dysfunction and oxidative stress in dopaminergic neurons, thereby promoting neurodegeneration in the substantia nigra. Experimental models further demonstrated that MMA exposure impairs ATP production and enhances reactive oxygen species generation, processes central to PD pathology. These findings highlight that the detrimental effects of MMA on neuronal survival are not limited to Alzheimer’s disease but may represent a broader mechanism underlying neurodegenerative diseases characterized by mitochondrial vulnerability.

Previous research has underscored the critical role of vitamin B12 in the development and maintenance of the nervous system, with a strong connection between vitamin B12 status and cognitive function. Several mechanisms have been proposed to explain how vitamin B12 deficiency may contribute to cognitive decline [42]. One key mechanism involves the conversion of vitamin B12 from its active form (methylcobalamin) to its inactive form (hydroxycobalamin).This conversion reduces the availability of methylcobalamin, impairing the enzyme activity of methionine synthase (MTR), which in turn inhibits the biosynthesis and regeneration of essential pathways [42]. Another potential neurotoxic mechanism is linked to vitamin B12 deficiency-induced myelin degradation and an imbalance of myelotoxic cytokines, which may lead to myelin loss and, ultimately, neuronal damage [43]. A recent two-year trial involving patients with mild cognitive impairment (MCI) found that high-dose supplementation with vitamin B6, folic acid, and vitamin B12 reduced the incidence of brain atrophy by 30% compared to a placebo. Among participants with elevated homocysteine (Hcy) levels, supplementation reduced the rate of brain atrophy by 53%, and changes in serum vitamin B12 or holo-TC levels were negatively correlated with atrophy rates [44]. These results suggest that brain atrophy measurements, along with domain-specific cognitive tests, may provide a more sensitive approach to exploring the relationship between vitamin B12 intake and cognitive function. However, a meta-analysis has cast doubt on the potential of B vitamins (B12, B6, and folic acid) as modifiable risk factors for slowing cognitive decline in older adults [45]. Despite evidence showing that these vitamins reduce Hcy levels, their supplementation did not significantly prevent or slow cognitive decline in individuals with mild cognitive impairment and elevated serum Hcy levels [46]. This finding indicates that, while B vitamins may reduce Hcy levels, they do not appear to offer substantial benefits in preventing or slowing cognitive decline in this population [47]. The results highlight a broader uncertainty regarding the role of vitamin B12 and other B vitamins in mitigating cognitive impairment, particularly in cognitively healthy middle-aged and older adults.

Our previous studies have found that functional cobalamin deficiency is more common than cobalamin deficiency and is associated with an increased risk of mortality in the general population [48]. Functional cobalamin deficiency, caused by cobalamin oxidation and inactivation, may progress more rapidly than typical cobalamin deficiency because it does not deplete the body’s cobalamin stores. Even higher and more frequent cobalamin doses have limited efficacy, suggesting decreased sensitivity to cobalamin therapy [49]. Furthermore, our previous studies have found that serum and dietary cobalamin levels were not associated with reduced mortality in adults with diabetes, whether using or not using metformin, whereas decreased cobalamin sensitivity was associated with all-cause and cardiac mortality, particularly in patients not using metformin [15]. MMA accumulation, but not serum or dietary vitamin B12, was associated with an increased risk of cardiovascular death in patients with coronary artery disease. This paradox may be related to decreased sensitivity to cobalamin [50,51]. Therefore, the term “decreased cobalamin sensitivity” is a more appropriate description of this condition than “functional cobalamin deficiency,” encompassing a broader description of the underlying pathophysiological processes that contribute to the reduced response to cobalamin therapy, including cobalamin absorption, trafficking, intracellular metabolism, and mitochondrial MMA metabolism [14,15,49,51,52]. Therefore, in addition to focusing on MMA, decreased cobalamin sensitivity should also be of greater concern for health promotion.

This study corroborates previous findings by demonstrating a significant association between serum vitamin B12, dietary vitamin B12, and supplemental vitamin B12 with cognitive impairment, but only in the context of high levels of MMA [53]. Elevated MMA levels were found to accelerate cognitive decline overall, with a doubling of MMA concentrations associated with a 60% increase in cognitive decline. Notably, MMA levels were negatively correlated with overall cognition and episodic memory, likely due to increased mitochondrial energy demands and the subsequent rise in intracellular free radicals, which could cause neuronal damage over time [54]. This aligns with earlier research showing that MMA, a marker of vitamin B12 deficiency, is associated with cognitive impairment. Further analysis revealed age-related differences in the association between MMA levels and cognitive impairment. A comprehensive study on a representative sample of the general population found significantly elevated serum MMA concentrations in individuals aged 18–20 years, while another study focusing on children aged 3–16 years reported a relationship between elevated MMA levels and lower cognitive scores [55]. Elevated MMA levels were found to accelerate cognitive decline overall, with a doubling of MMA concentrations associated with a 60% increase in cognitive decline [56]. Notably, MMA levels were negatively correlated with overall cognition and episodic memory, likely due to increased mitochondrial energy demands and the subsequent rise in intracellular free radicals, which could cause neuronal damage over time [57]. Research has shown an association between elevated serum MMA levels and cognitive impairment in patients with chronic kidney disease (CKD) [58]. Epidemiological studies consistently report that patients with CKD, regardless of disease stage, face a significantly higher risk of cognitive decline and dementia compared to non-CKD individuals [59]. This relationship is believed to be closely linked to the loss of renal function. The impaired excretory capacity of the kidneys in patients with CKD leads to the accumulation of MMA, urea, and other uremic toxins, which may indirectly influence cognitive function. Neuroimaging studies have further revealed that cognitive impairment in patients with CKD exhibiting lower eGFR affects multiple brain regions [60]. This impairment damages various cortical areas, particularly the frontal lobe, and also impacts subcortical neuronal regulation, including adrenergic neurons in the midbrain and cholinergic neurons in the Meynert basal ganglia. These findings underscore the mediating role of eGFR in predicting cognitive decline [61]. Notably, the synergistic interaction between neurodegenerative and vascular damage in the brain likely contributes to cognitive deterioration in individuals with impaired kidney function. The study’s strength lies in its ability to clarify the relationship between MMA and cognitive impairment in a population-based setting.

To the best of our knowledge, this is one of the first studies to explore this association within a nationally representative cohort, and the observed link between MMA levels and cognitive performance remained significant even after adjusting for potential confounders such as age, sex, and race/ethnicity. Sensitivity analyses further enhance the robustness of the results. The DSST score exhibits high stability in predicting cognitive impairment. Compared with a recent study that assessed cognitive function using only the DSST [62], our study applied four validated tests (CERAD-IR, CERAD-DR, DSST, and AFT), enabling a more comprehensive evaluation. However, certain limitations should be noted. Firstly, the NHANES database includes cognitive assessments only for individuals aged 60 and above, leaving the relationship between MMA and cognitive decline in younger populations unexplored. While the study adjusted for numerous confounding factors, the possibility of residual confounders or unmeasured variables remains. Moreover, as data from the 2011–2014 NHANES cycle did not include Hcy measurements, the potential association between Hcy and cognitive function could not be examined in this study. Thirdly, Dietary vitamin B12 intake and supplement use were based on participants’ self-reports from a 24-hour dietary recall, which may not accurately reflect long-term intake and could be subject to recall bias. Future studies could incorporate other long-term nutrient intake assessment methods or combine them with biomarker measurements to provide a more objective evaluation of individual dietary cobalamin status. Fourthly, While the associations in our study are derived from participants enrolled up to a decade ago, this is unlikely to substantially limit the representativeness of the population. Our previous work has reported that serum MMA levels and clinical characteristics were comparable between participants from 1999–2000 and those from 2013–2014 [15]. Nevertheless, we agree that future studies using more recent datasets would be valuable to validate these findings. Finally, given the observational nature of the research, causal conclusions cannot be drawn. Nonetheless, the large, representative sample of older adults in the United States enhances the reliability of the findings and addresses many methodological concerns.

## Conclusion

This study shows a significant link between high MMA levels and decline cognitive performance. This link may stem from mitochondrial dysfunction, particularly impaired propionic acid metabolism, causing MMA dysmetabolism, thus further impairing cognition. The study’s findings underscore MMA’s potential role in chronic cognitive decline.

## Supporting information

S1 Fig A restricted cubic spline analysis for the association between methylmalonic acid and decreased cognitive performance. (a) Response variable for DSST. MMA had a non-linear correlation with DSST. The X axis represents the log2 -MMA level, while the Y axis refers to the predicted cognitive function. (b) Response variable for AFT. MMA and AFT had a correlation that was not linear. (c) Response variable for CERAD-IR. MMA and CERAD-IR had a correlation that was not linear. (d) Response variable for CERAD-DR. MMA and CERAD-DR had a correlation that was not linear. Abbreviations: DSST, Digit Symbol Substitution Test; AFT, Animal Fluency test; CERAD, Consortium to Establish a Registry for Alzheimer’s Disease, CERAD-IR, CERAD immediate recall; CERAD-DR, CERAD delayed recall.
(DOCX)

S1 Table The Relationship between Methylmalonic Acid and Cognitions in NHANES 2011–2014. Calculated using linear regression. Abbreviations: DSST, Digit Symbol Substitution Test; AFT, Animal Fluency test; CERAD, Consortium to Establish a Registry for Alzheimer’s Disease. Model 1, adjusted for age (years, continuous), sex (female or male), and race/ethnicity (non-Hispanic white, black, Hispanic-Mexican, or other). Model 2, additionally adjusted for education level (less than high school, high school graduate, more than high school), smoking status (never, former, current), meeting recommended volume of physical activity (no/yes), alcohol consumption (male ≥ 20g/day, and female ≥ 10g/day), body mass index (kg/m2, continuous), systolic blood pressure (mmHg, continuous), the ratio of high-density lipoprotein to total cholesterol (ratio, continuous), type 2 diabetes (no/yes), stroked (no/yes), estimated glomerular filtration rate (≥ 60mL/min/1.73m², and <60 mL/min/1.73m²). Model 3, additionally adjusted for serum vitamin B12 (pmol/L, continuous). β* Unweight.
(DOCX)

S2 Table The Relationship between Serum Vitamin B12 and Cognitions in NHANES 2011–2014. Calculated using binary logistic regression. Ref, treating the bottom group (the lower than 250 (pmol/L) of Serum vitamin B12) as the reference. Abbreviations: CI, confidence interval; OR, odds ratio; DSST, Digit Symbol Substitution Test; AFT, Animal Fluency test; CERAD, Consortium to Establish a Registry for Alzheimer’s Disease. Model 1, adjusted for age (years, continuous), sex (female or male), and race/ethnicity (non-Hispanic white, black, Hispanic-Mexican, or other). Model 2, additionally adjusted for education level (less than high school, high school graduate, more than high school), smoking status (never, former, current), meeting recommended volume of physical activity (no/yes), alcohol consumption (male ≥ 20g/day, and female ≥ 10g/day), body mass index (kg/m2, continuous), systolic blood pressure (mmHg, continuous), the ratio of high-density lipoprotein to total cholesterol (ratio, continuous), type 2 diabetes (no/yes), stroked (no/yes), estimated glomerular filtration rate (≥ 60mL/min/1.73m², and <60 mL/min/1.73m²). Model 3, additionally adjusted for serum vitamin B12 (pmol/L, continuous). *P < 0.05, **P < 0.001.
(DOCX)

S3 Table The Relationship between B12 Intake from Foods and Cognitions in NHANES 2011–2014. Calculated using binary logistic regression. Ref, treating the bottom group (the lowest triples of B12 intake from foods) as the reference. Abbreviations: CI, confidence interval; OR, odds ratio.; DSST, Digit Symbol Substitution Test; AFT, Animal Fluency test; CERAD, Consortium to Establish a Registry for Alzheimer’s Disease. Model 1, adjusted for age (years, continuous), sex (female or male), and race/ethnicity (non-Hispanic white, black, Hispanic-Mexican, or other). Model 2, additionally adjusted for education level (less than high school, high school graduate, more than high school), smoking status (never, former, current), meeting recommended volume of physical activity (no/yes), alcohol consumption (male ≥ 20g/day, and female ≥ 10g/day), body mass index (kg/m2, continuous), systolic blood pressure (mmHg, continuous), the ratio of high-density lipoprotein to total cholesterol (ratio, continuous), type 2 diabetes (no/yes), stroked (no/yes), estimated glomerular filtration rate (≥ 60mL/min/1.73m², and <60 mL/min/1.73m²). Model 3, additionally adjusted for serum vitamin B12 (pmol/L, continuous). ^*^P < 0.05, ^**^P < 0.001.
(DOCX)

S4 Table The Relationship between B12 Supplements and Cognitions in NHANES 2011–2014. Calculated using binary logistic regression. Ref, treating no B12 supplements as the reference. Abbreviations: CI, confidence interval; OR, odds ratio.; DSST, Digit Symbol Substitution Test; AFT, Animal Fluency test; CERAD, Consortium to Establish a Registry for Alzheimer’s Disease. Model 1, adjusted for age (years, continuous), sex (female or male), and race/ethnicity (non-Hispanic white, black, Hispanic-Mexican, or other). Model 2, additionally adjusted for education level (less than high school, high school graduate, more than high school), smoking status (never, former, current), meeting recommended volume of physical activity (no/yes), alcohol consumption (male ≥ 20g/day, and female ≥ 10g/day), body mass index (kg/m2, continuous), systolic blood pressure (mmHg, continuous), the ratio of high-density lipoprotein to total cholesterol (ratio, continuous), type 2 diabetes (no/yes), stroked (no/yes), estimated glomerular filtration rate (≥ 60mL/min/1.73m², and <60 mL/min/1.73m²). Model 3, additionally adjusted for serum vitamin B12 (pmol/L, continuous). *P < 0.05, **P < 0.001.
(DOCX)

S5 Table The Serum vitamin B12 subgroup analysis for the Relationship between Methylmalonic acid and Cognitions in NHANES 2011–2014. Calculated using binary logistic regression; Ref, treating the bottom group (the lowest quartile of MMA) as the reference; Abbreviations: CI, confidence interval; OR, odds ratio.; DSST, Digit Symbol Substitution Test; AFT, Animal Fluency test; CERAD, Consortium to Establish a Registry for Alzheimer’s Disease. Model 1, adjusted for age (years, continuous), sex (female or male), and race/ethnicity (non-Hispanic white, black, Hispanic-Mexican, or other). Model 2, additionally adjusted for education level (less than high school, high school graduate, more than high school), smoking status (never, former, current), meeting recommended volume of physical activity (no/yes), alcohol consumption (male ≥ 20g/day, and female ≥ 10g/day), body mass index (kg/m2, continuous), systolic blood pressure (mmHg, continuous), the ratio of high-density lipoprotein to total cholesterol (ratio, continuous), type 2 diabetes (no/yes), stroked (no/yes), estimated glomerular filtration rate (≥ 60mL/min/1.73m², and <60 mL/min/1.73m²). Model 3, additionally adjusted for serum vitamin B12 (pmol/L, continuous). *P < 0.05, **P < 0.001.
(DOCX)

S6 Table The B12 intake from foods subgroup analysis for the Relationship between Methylmalonic acid and Cognitions in NHANES 2011–2014. Calculated using binary logistic regression; Ref, treating the bottom group (the lowest quartile of MMA) as the reference; Abbreviations: CI, confidence interval; OR, odds ratio; DSST, Digit Symbol Substitution Test; AFT, Animal Fluency test; CERAD, Consortium to Establish a Registry for Alzheimer’s Disease. Model 1, adjusted for age (years, continuous), sex (female or male), and race/ethnicity (non-Hispanic white, black, Hispanic-Mexican, or other). Model 2, additionally adjusted for education level (less than high school, high school graduate, more than high school), smoking status (never, former, current), meeting recommended volume of physical activity (no/yes), alcohol consumption (male ≥ 20g/day, and female ≥ 10g/day), body mass index (kg/m2, continuous), systolic blood pressure (mmHg, continuous), the ratio of high-density lipoprotein to total cholesterol (ratio, continuous), type 2 diabetes (no/yes), stroked (no/yes), estimated glomerular filtration rate (≥ 60mL/min/1.73m², and <60 mL/min/1.73m²). Model 3, additionally adjusted for serum vitamin B12 (pmol/L, continuous). *P < 0.05, **P < 0.001.
(DOCX)

S7 Table The B12 supplements subgroup analysis for the Relationship between Methylmalonic acid and Cognitions in NHANES 2011–2014. Calculated using binary logistic regression; Ref, treating the bottom group (the lowest quartile of MMA) as the reference; Abbreviations: CI, confidence interval; OR, odds ratio; DSST, Digit Symbol Substitution Test; AFT, Animal Fluency test; CERAD, Consortium to Establish a Registry for Alzheimer’s Disease. Model 1, adjusted for age (years, continuous), sex (female or male), and race/ethnicity (non-Hispanic white, black, Hispanic-Mexican, or other). Model 2, additionally adjusted for education level (less than high school, high school graduate, more than high school), smoking status (never, former, current), meeting recommended volume of physical activity (no/yes), alcohol consumption (male ≥ 20g/day, and female ≥ 10g/day), body mass index (kg/m2, continuous), systolic blood pressure (mmHg, continuous), the ratio of high-density lipoprotein to total cholesterol (ratio, continuous), type 2 diabetes (no/yes), stroked (no/yes), estimated glomerular filtration rate (≥ 60mL/min/1.73m², and <60 mL/min/1.73m²). Model 3, additionally adjusted for serum vitamin B12 (pmol/L, continuous). *P < 0.05, **P < 0.001.
(DOCX)

S8 Table The sex subgroup analysis for the Relationship between Methylmalonic acid and Cognitions in NHANES 2011–2014. Calculated using binary logistic regression; Ref, treating the bottom group (the lowest quartile of MMA) as the reference; Abbreviations: CI, confidence interval; OR, odds ratio; DSST, Digit Symbol Substitution Test; AFT, Animal Fluency test; CERAD, Consortium to Establish a Registry for Alzheimer’s Disease. Model 1, adjusted for age (years, continuous), sex (female or male), and race/ethnicity (non-Hispanic white, black, Hispanic-Mexican, or other). Model 2, additionally adjusted for education level (less than high school, high school graduate, more than high school), smoking status (never, former, current), meeting recommended volume of physical activity (no/yes), alcohol consumption (male ≥ 20g/day, and female ≥ 10g/day), body mass index (kg/m2, continuous), systolic blood pressure (mmHg, continuous), the ratio of high-density lipoprotein to total cholesterol (ratio, continuous), type 2 diabetes (no/yes), stroked (no/yes), estimated glomerular filtration rate (≥ 60mL/min/1.73m², and <60 mL/min/1.73m²). Model 3, additionally adjusted for serum vitamin B12 (pmol/L, continuous). *P < 0.05, **P < 0.001.
(DOCX)

S9 Table The age-related subgroup analysis for the Relationship between Methylmalonic acid and Cognitions in NHANES 2011–2014. Calculated using binary logistic regression; Ref, treating the bottom group (the lowest quartile of MMA) as the reference; Abbreviations: CI, confidence interval; OR, odds ratio; DSST, Digit Symbol Substitution Test; AFT, Animal Fluency test; CERAD, Consortium to Establish a Registry for Alzheimer’s Disease. Model 1, adjusted for age (years, continuous), sex (female or male), and race/ethnicity (non-Hispanic white, black, Hispanic-Mexican, or other). Model 2, additionally adjusted for education level (less than high school, high school graduate, more than high school), smoking status (never, former, current), meeting recommended volume of physical activity (no/yes), alcohol consumption (male ≥ 20g/day, and female ≥ 10g/day), body mass index (kg/m2, continuous), systolic blood pressure (mmHg, continuous), the ratio of high-density lipoprotein to total cholesterol (ratio, continuous), type 2 diabetes (no/yes), stroked (no/yes), estimated glomerular filtration rate (≥ 60mL/min/1.73m², and <60 mL/min/1.73m²). Model 3, additionally adjusted for serum vitamin B12 (pmol/L, continuous). *P < 0.05, **P < 0.001.
(DOCX)

## Abbreviations

BMI: body mass index
BP: blood pressure
TC/HDL-C ratio, eGFR: estimated glomerular filtration rate
CHD: coronary heart disease;physical activity
NHANES: the National Health and Nutrition Examination Survey
MMA: methylmalonic acid
DSST: Digit Symbol Substitution Test
AFT: Animal Fluency test
CERAD: Consortium to Establish a Registry for Alzheimer’s Disease
CERAD-IR: CERAD, score immediate recall
CERAD-DR: CERAD, score delayed recall, Error bars are 95%CIs
PCC: propionyl-CoA carboxylase
PCCB: Propionyl-CoA Carboxylase Subunit Beta
MCEE: Methylmalonyl-CoA epimerase
MMUT: methylmalonyl CoA mutase
TCA cycle: tricarboxylic acid cycle
Adocbl: adenosylcobalamin
cbl: cobalamin
MMAA: Methylmalonic aciduria type A
MMAB: Methylmalonic Aciduria cblB Type
MMADHC: methylmalonic aciduria and homocystinuria type D
MMACHC: methylmalonic aciduria and homocystinuria type C protein
LMBRD1: Lysosomal Cobalamin Transport Escort Protein LMBD1
ABCD4: ATP Binding Cassette Subfamily D Member 4
TGFBR1: Transforming Growth Factor Beta Receptor 1
OPA1: Optic Atrophy 1
VDAC3: Voltage Dependent Anion Channel 3
BRP2: Dystrophin Related Protein 2
MRPL10: mitochondrial ribosomal protein L10.
