# Supplementary material for: Mitochondrial dysfunction-related metabolite methylmalonic acid is associated with decreased cognitive performance
Source: PLoS One. 2025 Oct 17;20(10):e0332987. doi: 10.1371/journal.pone.0332987 (PMC12533889; doi:10.1371/journal.pone.0332987)
Supplement: S1 Fig — (a) Response variable for DSST. MMA had a non-linear correlation with DSST. The X axis represents the log2 -MMA level, while the Y axis refers to the predicted cognitive function. (b) Response variable for AFT. MMA and AFT had a correlation that was not linear. (c) Response variable for CERAD-IR. MMA and CERAD-IR had a correlation that was not linear. (d) Response variable for CERAD-DR. MMA and CERAD-DR had a correlation that was not linear. Abbreviations: DSST, Digit Symbol Substitution Test; AFT, Animal Fluency test; CERAD, Consortium to Establish a Registry for Alzheimer’s Disease, CERAD-IR, CERAD immediate recall; CERAD-DR, CERAD delayed recall. (DOCX) [file pone.0332987.s001.docx]

**Figure S1 A restricted cubic spline analysis for the association between methylmalonic acid and decreased cognitive performance.**


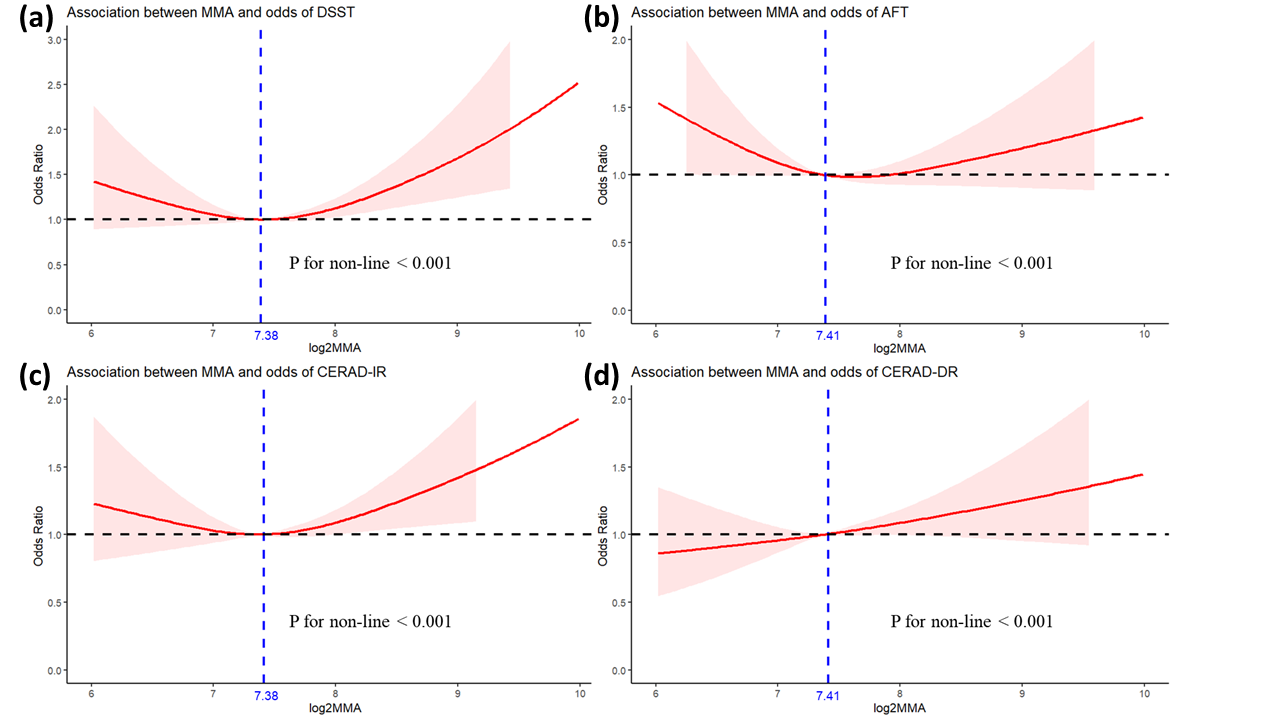


(a) Response variable for DSST. MMA had a non-linear correlation with DSST. The X axis represents the log2 -MMA level, while the Y axis refers to the predicted cognitive function. (b) Response variable for AFT. MMA and AFT had a correlation that was not linear. (c) Response variable for CERAD-IR. MMA and CERAD-IR had a correlation that was not linear. (d) Response variable for CERAD-DR. MMA and CERAD-DR had a correlation that was not linear.

Abbreviations: DSST, Digit Symbol Substitution Test; AFT, Animal Fluency test; CERAD, Consortium to Establish a Registry for Alzheimer’s Disease, CERAD-IR, CERAD immediate recall; CERAD-DR, CERAD delayed recall.
