# Supplementary material for: Mitochondrial dysfunction-related metabolite methylmalonic acid is associated with decreased cognitive performance
Source: PLoS One. 2025 Oct 17;20(10):e0332987. doi: 10.1371/journal.pone.0332987 (PMC12533889; doi:10.1371/journal.pone.0332987)
Supplement: S1 Table — Calculated using linear regression. Abbreviations: DSST, Digit Symbol Substitution Test; AFT, Animal Fluency test; CERAD, Consortium to Establish a Registry for Alzheimer’s Disease. Model 1, adjusted for age (years, continuous), sex (female or male), and race/ethnicity (non-Hispanic white, black, Hispanic-Mexican, or other). Model 2, additionally adjusted for education level (less than high school, high school graduate, more than high school), smoking status (never, former, current), meeting recommended volume of physical activity (no/yes), alcohol consumption (male ≥ 20g/day, and female ≥ 10g/day), body mass index (kg/m2, continuous), systolic blood pressure (mmHg, continuous), the ratio of high-density lipoprotein to total cholesterol (ratio, continuous), type 2 diabetes (no/yes), stroked (no/yes), estimated glomerular filtration rate (≥ 60mL/min/1.73m², and <60 mL/min/1.73m²). Model 3, additionally adjusted for serum vitamin B12 (pmol/L, continuous). β* Unweight. (DOCX) [file pone.0332987.s002.docx]

**Table S1. The Relationship between Methylmalonic Acid and Cognitions in NHANES 2011-2014**

|  | **Circulating methylmalonic acid (nmol/L)** | |
| --- | --- | --- |
|  | **β^*^** | *P* for trend |
| DSST scores |  |  |
| Crude | -0.17 | <0.001 |
| Model 1 | -0.12 | <0.001 |
| Model 2 | -0.07 | 0.015 |
| Model 3 | -0.07 | 0.01 |
| AFT |  |  |
| Crude | -0.02 | <0.001 |
| Model 1 | -0.02 | 0.001 |
| Model 2 | -0.01 | 0.187 |
| Model 3 | -0.01 | 0.114 |
| CERAD: score immediate recall |  |  |
| Crude | -0.03 | <0.001 |
| Model 1 | -0.02 | 0.002 |
| Model 2 | -0.01 | 0.111 |
| Model 3 | -0.01 | 0.091 |
| CERAD: score delayed recall |  |  |
| Crude | -0.02 | 0.001 |
| Model 1 | -0.01 | 0.091 |
| Model 2 | -0.02 | 0.487 |
| Model 3 | -0.02 | 0.413 |

Calculated using linear regression

Abbreviations: DSST, Digit Symbol Substitution Test; AFT, Animal Fluency test; CERAD, Consortium to Establish a Registry for Alzheimer’s Disease;

Model 1, adjusted for age (years, continuous), sex (female or male), and race/ethnicity (non-Hispanic white, black, Hispanic-Mexican, or other).

Model 2, additionally adjusted for education level (less than high school, high school graduate, more than high school), smoking status (never, former, current), meeting recommended volume of physical activity (no/yes), alcohol consumption (male ≥20g/day, and female ≥10g/day), body mass index (kg/m2, continuous), systolic blood pressure (mmHg, continuous), the ratio of high-density lipoprotein to total cholesterol (ratio, continuous), type 2 diabetes (no/yes), stroked (no/yes), estimated glomerular filtration rate (≥ 60mL/min/1.73m², and <60 mL/min/1.73m²).

Model 3, additionally adjusted for serum vitamin B12 (pmol/L, continuous).

β* Unweight
