# Supplementary material for: Mitochondrial dysfunction-related metabolite methylmalonic acid is associated with decreased cognitive performance
Source: PLoS One. 2025 Oct 17;20(10):e0332987. doi: 10.1371/journal.pone.0332987 (PMC12533889; doi:10.1371/journal.pone.0332987)
Supplement: S2 Table — Calculated using binary logistic regression. Ref, treating the bottom group (the lower than 250 (pmol/L) of Serum vitamin B12) as the reference. Abbreviations: CI, confidence interval; OR, odds ratio; DSST, Digit Symbol Substitution Test; AFT, Animal Fluency test; CERAD, Consortium to Establish a Registry for Alzheimer’s Disease. Model 1, adjusted for age (years, continuous), sex (female or male), and race/ethnicity (non-Hispanic white, black, Hispanic-Mexican, or other). Model 2, additionally adjusted for education level (less than high school, high school graduate, more than high school), smoking status (never, former, current), meeting recommended volume of physical activity (no/yes), alcohol consumption (male ≥ 20g/day, and female ≥ 10g/day), body mass index (kg/m2, continuous), systolic blood pressure (mmHg, continuous), the ratio of high-density lipoprotein to total cholesterol (ratio, continuous), type 2 diabetes (no/yes), stroked (no/yes), estimated glomerular filtration rate (≥ 60mL/min/1.73m², and <60 mL/min/1.73m²). Model 3, additionally adjusted for serum vitamin B12 (pmol/L, continuous). *P < 0.05, **P < 0.001. (DOCX) [file pone.0332987.s003.docx]

**Table S2.** **The Relationship between Serum Vitamin B12 and Cognitions in NHANES 2011-2014**

|  | **Serum vitamin B12, pmol/L** | | |
| --- | --- | --- | --- |
|  | **<250 (pmol/L) OR (95%CI)** | **>=250 (pmol/L) OR (95%CI)** | **>800 (pmol/L) OR (95%CI)** |
| DSST scores |  |  |  |
| Crude | 1.00(Ref.) | 0.71 (0.49 to 1.03) | 0.98 (0.55 to 1.74) |
| Model 1 | 1.00(Ref.) | 0.64 (0.43 to 0.95)^*^ | 0.70 (0.39 to 1.27) |
| Model 2 | 1.00(Ref.) | 0.71 (0.46 to 1.11) | 0.90 (0.49 to 1.65) |
| Model 3 | 1.00(Ref.) | 0.69 (0.44 to 1.10) | 0.80 (0.35 to 1.79) |
| AFT |  |  |  |
| Crude | 1.00(Ref.) | 0.94 (0.65 to 1.34) | 1.16 (0.75 to 1.80) |
| Model 1 | 1.00(Ref.) | 0.89 (0.60 to 1.32) | 0.91 (0.58 to 1.44) |
| Model 2 | 1.00(Ref.) | 0.96 (0.64 to 1.43) | 0.99 (0.58 to 1.70) |
| Model 3 | 1.00(Ref.) | 0.94 (0.66 to 1.35) | 0.91 (0.51 to 1.65) |
| CERAD: score immediate recall |  |  |  |
| Crude | 1.00(Ref.) | 0.85 (0.63 to 1.13) | 1.44 (1.00 to 2.06)^*^ |
| Model 1 | 1.00(Ref.) | 0.85 (0.64 to 1.13) | 1.48 (1.01 to 2.17)^*^ |
| Model 2 | 1.00(Ref.) | 0.92 (0.65 to 1.31) | 1.70 (1.08 to 2.67)^*^ |
| Model 3 | 1.00(Ref.) | 0.98 (0.70 to 1.37) | 2.29 (1.38 to 3.79)^**^ |
| CERAD: score delayed recall |  |  |  |
| Crude | 1.00(Ref.) | 0.13 (1.34 to 0.00) | 1.24 (0.80 to 1.94) |
| Model 1 | 1.00(Ref.) | 1.05 (0.83 to 1.33) | 1.23 (0.77 to 1.94) |
| Model 2 | 1.00(Ref.) | 1.12 (0.86 to 1.47) | 1.30 (0.79 to 2.14) |
| Model 3 | 1.00(Ref.) | 1.36 (1.03 to 1.80)^*^ | 2.99 (1.57 to 5.71)^**^ |

Calculated using binary logistic regression;

Ref, treating the bottom group (the lower than 250 (pmol/L) of Serum vitamin B12) as the reference;

Abbreviations: CI, confidence interval; OR, odds ratio; DSST, Digit Symbol Substitution Test; AFT, Animal Fluency test; CERAD, Consortium to Establish a Registry for Alzheimer’s Disease;

Model 1, adjusted for age (years, continuous), sex (female or male), and race/ethnicity (non-Hispanic white, black, Hispanic-Mexican, or other).

Model 2, additionally adjusted for education level (less than high school, high school graduate, more than high school), smoking status (never, former, current), meeting recommended volume of physical activity (no/yes), alcohol consumption (male ≥20g/day, and female ≥10g/day), body mass index (kg/m2, continuous), systolic blood pressure (mmHg, continuous), the ratio of high-density lipoprotein to total cholesterol (ratio, continuous), type 2 diabetes (no/yes), stroked (no/yes), estimated glomerular filtration rate (≥ 60mL/min/1.73m², and <60 mL/min/1.73m²).

Model 3, additionally adjusted for serum vitamin B12 (pmol/L, continuous).

^*^*P* < 0.05, ^**^*P*<0.001
