# Supplementary material for: Mitochondrial dysfunction-related metabolite methylmalonic acid is associated with decreased cognitive performance
Source: PLoS One. 2025 Oct 17;20(10):e0332987. doi: 10.1371/journal.pone.0332987 (PMC12533889; doi:10.1371/journal.pone.0332987)
Supplement: S3 Table — Calculated using binary logistic regression. Ref, treating the bottom group (the lowest triples of B12 intake from foods) as the reference. Abbreviations: CI, confidence interval; OR, odds ratio.; DSST, Digit Symbol Substitution Test; AFT, Animal Fluency test; CERAD, Consortium to Establish a Registry for Alzheimer’s Disease. Model 1, adjusted for age (years, continuous), sex (female or male), and race/ethnicity (non-Hispanic white, black, Hispanic-Mexican, or other). Model 2, additionally adjusted for education level (less than high school, high school graduate, more than high school), smoking status (never, former, current), meeting recommended volume of physical activity (no/yes), alcohol consumption (male ≥ 20g/day, and female ≥ 10g/day), body mass index (kg/m2, continuous), systolic blood pressure (mmHg, continuous), the ratio of high-density lipoprotein to total cholesterol (ratio, continuous), type 2 diabetes (no/yes), stroked (no/yes), estimated glomerular filtration rate (≥ 60mL/min/1.73m², and <60 mL/min/1.73m²). Model 3, additionally adjusted for serum vitamin B12 (pmol/L, continuous). *P < 0.05, **P < 0.001. (DOCX) [file pone.0332987.s004.docx]

**Table S3. The Relationship between** **B12 Intake from Foods and Cognitions in NHANES 2011-2014**

|  | **B12 intake from foods(μg/day)** | | |
| --- | --- | --- | --- |
|  | **Q1 OR (95%CI)** | **Q2 OR (95%CI)** | **Q3 OR (95%CI)** |
| DSST scores |  |  |  |
| Crude | 1.00(Ref.) | 0.57 (0.29 to 1.15) | 0.40 (0.21 to 0.77)^**^ |
| Model 1 | 1.00(Ref.) | 0.62 (0.32 to 1.18) | 0.47 (0.26 to 0.85)^*^ |
| Model 2 | 1.00(Ref.) | 0.66 (0.37 to 1.21) | 0.53 (0.28 to 1.01) |
| Model 3 | 1.00(Ref.) | 0.66 (0.36 to 1.21) | 0.54 (0.28 to 1.04) |
| AFT |  |  |  |
| Crude | 1.00(Ref.) | 0.53 (0.27 to 1.02) | 0.50 (0.28to 0.87)^*^ |
| Model 1 | 1.00(Ref.) | 0.54 (0.29 to 1.02) | 0.56 (0.32 to 0.98)* |
| Model 2 | 1.00(Ref.) | 0.61 (0.32 to 1.16) | 0.69 (0.36 to 1.30) |
| Model 3 | 1.00(Ref.) | 0.61 (0.32 to 1.16) | 0.69 (0.37 to 1.30) |
| CERAD: score immediate recall |  |  |  |
| Crude | 1.00(Ref.) | 1.03 (0.57 to 1.86) | 1.09 (0.71 to 1.67) |
| Model 1 | 1.00(Ref.) | 1.06 (0.57 to 1.96) | 1.10 (0.71 to 1.7) |
| Model 2 | 1.00(Ref.) | 1.28 (0.65 to 2.52) | 1.53 (0.96 to 2.46) |
| Model 3 | 1.00(Ref.) | 1.28 (0.65 to 2.51) | 1.53 (0.95 to 2.45) |
| CERAD: score delayed recall |  |  |  |
| Crude | 1.00(Ref.) | 1.12 (0.64 to 1.94) | 1.17 (0.67 to 2.07) |
| Model 1 | 1.00(Ref.) | 1.14 (0.68 to 1.91) | 1.25 (0.72 to 2.17) |
| Model 2 | 1.00(Ref.) | 1.39 (0.75 to 2.55) | 1.52 (0.84 to 2.74) |
| Model 3 | 1.00(Ref.) | 1.38 (0.75 to 2.54) | 1.52 (0.84 to 2.73) |

Calculated using binary logistic regression;

Ref, treating the bottom group (the lowest triples of B12 intake from foods) as the reference;

Abbreviations: CI, confidence interval; OR, odds ratio.; DSST, Digit Symbol Substitution Test; AFT, Animal Fluency test; CERAD, Consortium to Establish a Registry for Alzheimer’s Disease;

Model 1, adjusted for age (years, continuous), sex (female or male), and race/ethnicity (non-Hispanic white, black, Hispanic-Mexican, or other).

Model 2, additionally adjusted for education level (less than high school, high school graduate, more than high school), smoking status (never, former, current), meeting recommended volume of physical activity (no/yes), alcohol consumption (male ≥20g/day, and female ≥10g/day), body mass index (kg/m2, continuous), systolic blood pressure (mmHg, continuous), the ratio of high-density lipoprotein to total cholesterol (ratio, continuous), type 2 diabetes (no/yes), stroked (no/yes), estimated glomerular filtration rate (≥ 60mL/min/1.73m², and <60 mL/min/1.73m²).

Model 3, additionally adjusted for serum vitamin B12 (pmol/L, continuous).

^*^*P* < 0.05, ^**^*P*<0.001
