# Supplementary material for: Mitochondrial dysfunction-related metabolite methylmalonic acid is associated with decreased cognitive performance
Source: PLoS One. 2025 Oct 17;20(10):e0332987. doi: 10.1371/journal.pone.0332987 (PMC12533889; doi:10.1371/journal.pone.0332987)
Supplement: S4 Table — Calculated using binary logistic regression. Ref, treating no B12 supplements as the reference. Abbreviations: CI, confidence interval; OR, odds ratio.; DSST, Digit Symbol Substitution Test; AFT, Animal Fluency test; CERAD, Consortium to Establish a Registry for Alzheimer’s Disease. Model 1, adjusted for age (years, continuous), sex (female or male), and race/ethnicity (non-Hispanic white, black, Hispanic-Mexican, or other). Model 2, additionally adjusted for education level (less than high school, high school graduate, more than high school), smoking status (never, former, current), meeting recommended volume of physical activity (no/yes), alcohol consumption (male ≥ 20g/day, and female ≥ 10g/day), body mass index (kg/m2, continuous), systolic blood pressure (mmHg, continuous), the ratio of high-density lipoprotein to total cholesterol (ratio, continuous), type 2 diabetes (no/yes), stroked (no/yes), estimated glomerular filtration rate (≥ 60mL/min/1.73m², and <60 mL/min/1.73m²). Model 3, additionally adjusted for serum vitamin B12 (pmol/L, continuous). *P < 0.05, **P < 0.001. (DOCX) [file pone.0332987.s005.docx]

**Table S4. The Relationship between B12 Supplements and Cognitions in NHANES 2011-2014**

|  | **B12 supplements** | |
| --- | --- | --- |
|  | **No** | **Yes** |
|  | **OR (95%CI)** | **OR (95%CI)** |
| DSST scores |  |  |
| Crude | 1.00(Ref.) | 0.63 (0.49 to 0.81)^**^ |
| Model 1 | 1.00(Ref.) | 0.61 (0.45 to 0.83)^**^ |
| Model 2 | 1.00(Ref.) | 0.75 (0.54 to 1.03) |
| Model 3 | 1.00(Ref.) | 0.71 (0.52 to 0.96)^*^ |
| AFT |  |  |
| Crude | 1.00(Ref.) | 0.87 (0.71 to 1.06) |
| Model 1 | 1.00(Ref.) | 0.86 (0.68 to 1.09) |
| Model 2 | 1.00(Ref.) | 0.99 (0.76 to 1.29) |
| Model 3 | 1.00(Ref.) | 0.98 (0.76 to 1.25) |
| CERAD: score immediate recall |  |  |
| Crude | 1.00(Ref.) | 0.93 (0.76 to 1.13) |
| Model 1 | 1.00(Ref.) | 0.90 (0.72 to 1.11) |
| Model 2 | 1.00(Ref.) | 1.00 (0.78 to 1.29) |
| Model 3 | 1.00(Ref.) | 1.00 (0.79 to 1.28) |
| Model4 | 1.00(Ref.) | 0.98 (0.77 to 1.24) |
| CERAD: score delayed recall |  |  |
| Crude | 1.00(Ref.) | 0.95 (0.76 to 1.19) |
| Model 1 | 1.00(Ref.) | 0.88 (0.71 to 1.10) |
| Model 2 | 1.00(Ref.) | 0.95 (0.73 to 1.23) |
| Model 3 | 1.00(Ref.) | 0.98 (0.74 to 1.31) |

Calculated using binary logistic regression;

Ref, treating no B12 supplements as the reference;

Abbreviations: CI, confidence interval; OR, odds ratio.; DSST, Digit Symbol Substitution Test; AFT, Animal Fluency test; CERAD, Consortium to Establish a Registry for Alzheimer’s Disease;

Model 1, adjusted for age (years, continuous), sex (female or male), and race/ethnicity (non-Hispanic white, black, Hispanic-Mexican, or other).

Model 2, additionally adjusted for education level (less than high school, high school graduate, more than high school), smoking status (never, former, current), meeting recommended volume of physical activity (no/yes), alcohol consumption (male ≥20g/day, and female ≥10g/day), body mass index (kg/m2, continuous), systolic blood pressure (mmHg, continuous), the ratio of high-density lipoprotein to total cholesterol (ratio, continuous), type 2 diabetes (no/yes), stroked (no/yes), estimated glomerular filtration rate (≥ 60mL/min/1.73m², and <60 mL/min/1.73m²).

Model 3, additionally adjusted for serum vitamin B12 (pmol/L, continuous).

^*^*P* < 0.05, ^**^*P*<0.001
