# Supplementary material for: Mitochondrial dysfunction-related metabolite methylmalonic acid is associated with decreased cognitive performance
Source: PLoS One. 2025 Oct 17;20(10):e0332987. doi: 10.1371/journal.pone.0332987 (PMC12533889; doi:10.1371/journal.pone.0332987)
Supplement: S5 Table — Calculated using binary logistic regression; Ref, treating the bottom group (the lowest quartile of MMA) as the reference; Abbreviations: CI, confidence interval; OR, odds ratio.; DSST, Digit Symbol Substitution Test; AFT, Animal Fluency test; CERAD, Consortium to Establish a Registry for Alzheimer’s Disease. Model 1, adjusted for age (years, continuous), sex (female or male), and race/ethnicity (non-Hispanic white, black, Hispanic-Mexican, or other). Model 2, additionally adjusted for education level (less than high school, high school graduate, more than high school), smoking status (never, former, current), meeting recommended volume of physical activity (no/yes), alcohol consumption (male ≥ 20g/day, and female ≥ 10g/day), body mass index (kg/m2, continuous), systolic blood pressure (mmHg, continuous), the ratio of high-density lipoprotein to total cholesterol (ratio, continuous), type 2 diabetes (no/yes), stroked (no/yes), estimated glomerular filtration rate (≥ 60mL/min/1.73m², and <60 mL/min/1.73m²). Model 3, additionally adjusted for serum vitamin B12 (pmol/L, continuous). *P < 0.05, **P < 0.001. (DOCX) [file pone.0332987.s006.docx]

**Table S5. The** **Serum vitamin B12 subgroup analysis for the Relationship between Methylmalonic acid and Cognitions in NHANES 2011-2014**

|  | **Circulating methylmalonic acid (nmol/L)** | | | |  |
| --- | --- | --- | --- | --- | --- |
|  | **Q1 OR (95%CI)** | **Q2 OR (95%CI)** | **Q3 OR (95%CI)** | **Q4 OR (95%CI)** |  |
|  |  |  |  |  |  |
| **Serum vitamin B12>400(pmol/L)** |  |  |  |  |  |
| DSST scores |  |  |  |  |  |
| Crude | 1.00(Ref.) | 0.84 (0.56 to 1.25) | 1.11 (0.73 to 1.68) | 2.72 (1.84 to 4.04)^**^ |  |
| Model 1 | 1.00(Ref.) | 0.84 (0.55 to 1.28) | 1.04 (0.64 to 1.69) | 2.64 (1.44 to 4.83)^**^ |  |
| Model 2 | 1.00(Ref.) | 0.79 (0.48 to 1.30) | 0.91 (0.54 to 1.53) | 2.03 (1.06 to 3.88)* |  |
| Model 3 | 1.00(Ref.) | 0.86 (0.52 to 1.41) | 1.02 (0.63 to 1.67) | 2.29 (1.19 to 4.42)^*^ |  |
| AFT |  |  |  |  |  |
| Model 1 | 1.00(Ref.) | 1.09 (0.65 to 1.82) | 1.49 (0.97 to 2.30) | 2.38 (1.32 to 4.30)^*^ |  |
| Model 2 | 1.00(Ref.) | 1.07 (0.63 to 1.83) | 1.32 (0.79 to 2.22) | 1.93 (1.01 to 3.70) |  |
| Model 3 | 1.00(Ref.) | 1.02 (0.57 to 1.82) | 1.14 (0.68 to 1.93) | 1.45 (0.74 to 2.84) |  |
| Model4 | 1.00(Ref.) | 1.01 (0.57 to 1.80) | 1.13 (0.69 to 1.87) | 1.44 (0.77 to 2.71) |  |
| CERAD: score immediate recall |  |  |  |  |  |
| Crude | 1.00(Ref.) | 1.15 (0.62 to 2.12) | 1.88 (1.17 to 3.05)^*^ | 2.49 (1.72 to 3.62)^**^ |  |
| Model 1 | 1.00(Ref.) | 1.07 (0.55 to 2.07) | 1.48 (0.83 to 2.66) | 1.73 (1.06 to 2.82)^*^ |  |
| Model 2 | 1.00(Ref.) | 0.96 (0.47 to 1.97) | 1.09 (0.56 to 2.12) | 1.09 (0.59 to 2.05) |  |
| Model 3 | 1.00(Ref.) | 0.96 (0.46 to 1.97) | 1.10 (0.56 to 2.14) | 1.09 (0.58 to 2.05) |  |
| CERAD: score delayed recall |  |  |  |  |  |
| Crude | 1.00(Ref.) | 1.13 (0.60 to 2.13) | 2.13 (1.34 to 3.38)^**^ | 2.78 (1.85 to 4.17)^**^ |  |
| Model 1 | 1.00(Ref.) | 0.97 (0.53 to 1.79) | 1.46 (0.87 to 2.44) | 1.59 (1.06 to 2.38)^*^ |  |
| Model 2 | 1.00(Ref.) | 0.99 (0.50 to 1.93) | 1.35 (0.75 to 2.43) | 1.26 (0.76 to 2.09) |  |
| Model 3 | 1.00(Ref.) | 1.00 (0.50 to 1.98) | 1.35 (0.74 to 2.46) | 1.26 (0.76 to 2.09) |  |
| **Serum vitamin B12≤400(pmol/L)** |  |  |  |  |  |
| Variables |  |  |  |  |  |
| DSST scores |  |  |  |  |  |
| Crude | 1.00(Ref.) | 0.74 (0.44 to 1.25) | 0.95 (0.59 to 1.52) | 1.65 (1.09 to 2.51)^*^ |  |
| Model 1 | 1.00(Ref.) | 0.64 (0.36 to 1.11) | 0.88 (0.53 to 1.46) | 1.34 (0.86 to 2.11) |  |
| Model 2 | 1.00(Ref.) | 0.81 (0.41 to 1.60) | 0.98 (0.49 to 1.96) | 1.17 (0.59 to 2.35) |  |
| Model 3 | 1.00(Ref.) | 0.81 (0.42 to 1.59) | 0.97 (0.49 to 1.91) | 1.00 (0.49 to 2.01) |  |
| AFT |  |  |  |  |  |
| Crude | 1.00(Ref.) | 0.81 (0.38 to 1.76) | 0.82 (0.46 to 1.47) | 1.49 (0.88 to 2.50) |  |
| Model 1 | 1.00(Ref.) | 0.74 (0.33 to 1.67) | 0.75 (0.43 to 1.29) | 1.21 (0.73 to 2.01) |  |
| Model 2 | 1.00(Ref.) | 0.80 (0.35 to 1.87) | 0.71 (0.37 to 1.37) | 1.04 (0.56 to 1.96) |  |
| Model 3 | 1.00(Ref.) | 0.81 (0.35 to 1.87) | 0.71 (0.37 to 1.37) | 0.98 (0.50 to 1.92) |  |
| CERAD: score immediate recall |  |  |  |  |  |
| Crude | 1.00(Ref.) | 1.04 (0.60 to 1.79) | 1.21 (0.79 to 1.86) | 2.02 (1.39 to 2.92)^**^ |  |
| Model 1 | 1.00(Ref.) | 0.85 (0.46 to 1.58) | 0.96 (0.60 to 1.52) | 1.45 (0.87 to 2.42) |  |
| Model 2 | 1.00(Ref.) | 1.02 (0.51 to 2.04) | 1.09 (0.63 to 1.88) | 1.51 (0.85 to 2.68) |  |
| Model 3 | 1.00(Ref.) | 1.02 (0.52 to 2.01) | 1.08 (0.62 to 1.88) | 1.53 (0.82 to 2.86) |  |
| CERAD: score delayed recall |  |  |  |  |  |
| Crude | 1.00(Ref.) | 1.44 (0.83 to 2.49) | 1.44 (0.82 to 2.55) | 2.01 (1.32 to 3.07)^**^ |  |
| Model 1 | 1.00(Ref.) | 1.19 (0.64 to 2.21) | 1.11 (0.59 to 2.08) | 1.41 (0.79 to 2.51) |  |
| Model 2 | 1.00(Ref.) | 1.22 (0.66 to 2.27) | 1.04 (0.49 to 2.21) | 1.27 (0.68 to 2.36) |  |
| Model 3 | 1.00(Ref.) | 1.20 (0.64 to 2.25) | 1.03 (0.47 to 2.23) | 1.36 (0.70 to 2.64) |  |

Calculated using binary logistic regression;

Ref, treating the bottom group (the lowest quartile of MMA) as the reference;

Abbreviations: CI, confidence interval; OR, odds ratio.; DSST, Digit Symbol Substitution Test; AFT, Animal Fluency test; CERAD, Consortium to Establish a Registry for Alzheimer’s Disease;

Model 1, adjusted for age (years, continuous), sex (female or male), and race/ethnicity (non-Hispanic white, black, Hispanic-Mexican, or other).

Model 2, additionally adjusted for education level (less than high school, high school graduate, more than high school), smoking status (never, former, current), meeting recommended volume of physical activity (no/yes), alcohol consumption (male ≥20g/day, and female ≥10g/day), body mass index (kg/m2, continuous), systolic blood pressure (mmHg, continuous), the ratio of high-density lipoprotein to total cholesterol (ratio, continuous), type 2 diabetes (no/yes), stroked (no/yes), estimated glomerular filtration rate (≥ 60mL/min/1.73m², and <60 mL/min/1.73m²).

Model 3, additionally adjusted for serum vitamin B12 (pmol/L, continuous).

^*^*P* < 0.05, ^**^*P*<0.001
