# Supplementary material for: Mitochondrial dysfunction-related metabolite methylmalonic acid is associated with decreased cognitive performance
Source: PLoS One. 2025 Oct 17;20(10):e0332987. doi: 10.1371/journal.pone.0332987 (PMC12533889; doi:10.1371/journal.pone.0332987)
Supplement: S6 Table — Calculated using binary logistic regression; Ref, treating the bottom group (the lowest quartile of MMA) as the reference; Abbreviations: CI, confidence interval; OR, odds ratio; DSST, Digit Symbol Substitution Test; AFT, Animal Fluency test; CERAD, Consortium to Establish a Registry for Alzheimer’s Disease. Model 1, adjusted for age (years, continuous), sex (female or male), and race/ethnicity (non-Hispanic white, black, Hispanic-Mexican, or other). Model 2, additionally adjusted for education level (less than high school, high school graduate, more than high school), smoking status (never, former, current), meeting recommended volume of physical activity (no/yes), alcohol consumption (male ≥ 20g/day, and female ≥ 10g/day), body mass index (kg/m2, continuous), systolic blood pressure (mmHg, continuous), the ratio of high-density lipoprotein to total cholesterol (ratio, continuous), type 2 diabetes (no/yes), stroked (no/yes), estimated glomerular filtration rate (≥ 60mL/min/1.73m², and <60 mL/min/1.73m²). Model 3, additionally adjusted for serum vitamin B12 (pmol/L, continuous). *P < 0.05, **P < 0.001. (DOCX) [file pone.0332987.s007.docx]

**Table S6. The B12 intake from foods subgroup analysis for the Relationship between Methylmalonic acid and Cognitions in NHANES 2011-2014**

|  | **Circulating methylmalonic acid (nmol/L)** | | | |  |
| --- | --- | --- | --- | --- | --- |
|  | **Q1 OR (95%CI)** | **Q2 OR (95%CI)** | **Q3 OR (95%CI)** | **Q4 OR (95%CI)** |  |
|  |  |  |  |  |  |
| **B12 intake from foods>5.07(μg/day)** |  |  |  |  |  |
| DSST score |  |  |  |  |  |
| Crude | 1.00(Ref.) | 0.84 (0.52 to 1.38) | 1.61 (1.05 to 2.46)^*^ | 2.91 (1.55 to 5.47)^**^ |  |
| Model 1 | 1.00(Ref.) | 0.85 (0.51 to 1.43) | 1.53 (0.90 to 2.58) | 2.55 (1.36 to 4.80)^*^ |  |
| Model 2 | 1.00(Ref.) | 0.81 (0.41 to 1.61) | 1.37 (0.65 to 2.93) | 2.03 (1.00 to 4.14) |  |
| Model 3 | 1.00(Ref.) | 0.79 (0.41 to 1.51) | 1.74 (0.86 to 3.49) | 2.77 (1.37 to 5.59)^*^ |  |
| AFT |  |  |  |  |  |
| Crude | 1.00(Ref.) | 1.12 (0.53 to 2.35) | 1.29 (0.68 to 2.44) | 2.34 (1.19 to 4.58)^*^ |  |
| Model 1 | 1.00(Ref.) | 1.08 (0.52 to 2.25) | 1.04 (0.56 to 1.93) | 1.64 (0.80 to 3.37) |  |
| Model 2 | 1.00(Ref.) | 1.11 (0.52 to 2.38) | 0.85 (0.41 to 1.73) | 1.21 (0.59 to 2.49) |  |
| Model 3 | 1.00(Ref.) | 1.13 (0.53 to 2.43) | 0.90 (0.42 to 1.93) | 1.31 (0.60 to 2.88) |  |
| CERAD: score immediate recall |  |  |  |  |  |
| Crude | 1.00(Ref.) | 1.03 (0.56 to 1.87) | 1.68 (0.97 to 2.90) | 2.64 (1.63 to 4.30)^**^ |  |
| Model 1 | 1.00(Ref.) | 0.87 (0.48 to 1.58) | 1.14 (0.60 to 2.16) | 1.49 (0.88 to 2.49) |  |
| Model 2 | 1.00(Ref.) | 0.92 (0.46 to 1.82) | 1.07 (0.50 to 2.29) | 1.22 (0.62 to 2.37) |  |
| Model 3 | 1.00(Ref.) | 0.91 (0.46 to 1.80) | 1.01 (0.47 to 2.17) | 1.12 (0.57 to 2.21) |  |
| CERAD: score delayed recall |  |  |  |  |  |
| Crude | 1.00(Ref.) | 1.38 (0.71 to 2.68) | 2.11 (1.14 to 3.89)^*^ | 2.42 (1.35 to 4.35)^**^ |  |
| Model 1 | 1.00(Ref.) | 1.11 (0.56 to 2.20) | 1.32 (0.65 to 2.71) | 1.20 (0.64 to 2.22) |  |
| Model 2 | 1.00(Ref.) | 1.27 (0.66 to 2.45) | 1.29 (0.60 to 2.76) | 0.89 (0.42 to 1.90) |  |
| Model 3 | 1.00(Ref.) | 1.22 (0.64 to 2.34) | 1.15 (0.52 to 2.52) | 0.76 (0.36 to 1.60) |  |
| **B12 intake from foods≤5.07(μg/day)** |  |  |  |  |  |
| Variables |  |  |  |  |  |
| DSST score |  |  |  |  |  |
| Crude | 1.00(Ref.) | 0.79 (0.52 to 1.20) | 0.84 (0.55 to 1.28) | 1.77 (1.25 to 2.52)^**^ |  |
| Model 1 | 1.00(Ref.) | 0.72 (0.46 to 1.12) | 0.83 (0.51 to 1.35) | 1.72 (1.07 to 2.77)^*^ |  |
| Model 2 | 1.00(Ref.) | 0.73 (0.42 to 1.28) | 0.76 (0.43 to 1.34) | 1.22 (0.68 to 2.20) |  |
| Model 3 | 1.00(Ref.) | 0.76 (0.43 to 1.34) | 0.78 (0.44 to 1.40) | 1.23 (0.66 to 2.30) |  |
| AFT |  |  |  |  |  |
| Crude | 1.00(Ref.) | 0.92 (0.58 to 1.45) | 1.03 (0.62 to 1.73) | 1.56 (0.92 to 2.66) |  |
| Model 1 | 1.00(Ref.) | 0.86 (0.54 to 1.36) | 1.00 (0.57 to 1.73) | 1.40 (0.79 to 2.46) |  |
| Model 2 | 1.00(Ref.) | 0.78 (0.49 to 1.25) | 0.88 (0.51 to 1.52) | 1.02 (0.57 to 1.83) |  |
| Model 3 | 1.00(Ref.) | 0.78 (0.48 to 1.25) | 0.88 (0.50 to 1.53) | 1.01 (0.56 to 1.84) |  |
| CERAD: score immediate recall |  |  |  |  |  |
| Crude | 1.00(Ref.) | 1.15 (0.71 to 1.85) | 1.40 (0.84 to 2.34) | 1.95 (1.44 to 2.65)^**^ |  |
| Model 1 | 1.00(Ref.) | 1.03 (0.62 to 1.72) | 1.20 (0.74 to 1.95) | 1.53 (1.09 to 2.15)^*^ |  |
| Model 2 | 1.00(Ref.) | 0.99 (0.60 to 1.64) | 1.02 (0.64 to 1.64) | 1.12 (0.79 to 1.59) |  |
| Model 3 | 1.00(Ref.) | 1.01 (0.61 to 1.69) | 1.07 (0.67 to 1.71) | 1.20 (0.84 to 1.70) |  |
| CERAD: score delayed recall |  |  |  |  |  |
| Crude | 1.00(Ref.) | 1.21 (0.71 to 2.04) | 1.57 (0.99 to 2.50) | 2.28 (1.59 to 3.27)^*^ |  |
| Model 1 | 1.00(Ref.) | 1.07 (0.62 to 1.82) | 1.30 (0.83 to 2.04) | 1.71 (1.12 to 2.59) |  |
| Model 2 | 1.00(Ref.) | 1.05 (0.59 to 1.88) | 1.20 (0.70 to 2.04) | 1.47 (0.90 to 2.41) |  |
| Model 3 | 1.00(Ref.) | 1.08 (0.60 to 1.94) | 1.23 (0.73 to 2.08) | 1.51 (0.93 to 2.45) |  |

Calculated using binary logistic regression;

Ref, treating the bottom group (the lowest quartile of MMA) as the reference;

Abbreviations: CI, confidence interval; OR, odds ratio; DSST, Digit Symbol Substitution Test; AFT, Animal Fluency test; CERAD, Consortium to Establish a Registry for Alzheimer’s Disease;

Model 1, adjusted for age (years, continuous), sex (female or male), and race/ethnicity (non-Hispanic white, black, Hispanic-Mexican, or other).

Model 2, additionally adjusted for education level (less than high school, high school graduate, more than high school), smoking status (never, former, current), meeting recommended volume of physical activity (no/yes), alcohol consumption (male ≥20g/day, and female ≥10g/day), body mass index (kg/m2, continuous), systolic blood pressure (mmHg, continuous), the ratio of high-density lipoprotein to total cholesterol (ratio, continuous), type 2 diabetes (no/yes), stroked (no/yes), estimated glomerular filtration rate (≥ 60mL/min/1.73m², and <60 mL/min/1.73m²).

Model 3, additionally adjusted for serum vitamin B12 (pmol/L, continuous).

^*^*P* < 0.05, ^**^*P*<0.001
