# Supplementary material for: Mitochondrial dysfunction-related metabolite methylmalonic acid is associated with decreased cognitive performance
Source: PLoS One. 2025 Oct 17;20(10):e0332987. doi: 10.1371/journal.pone.0332987 (PMC12533889; doi:10.1371/journal.pone.0332987)
Supplement: S7 Table — Calculated using binary logistic regression; Ref, treating the bottom group (the lowest quartile of MMA) as the reference; Abbreviations: CI, confidence interval; OR, odds ratio; DSST, Digit Symbol Substitution Test; AFT, Animal Fluency test; CERAD, Consortium to Establish a Registry for Alzheimer’s Disease. Model 1, adjusted for age (years, continuous), sex (female or male), and race/ethnicity (non-Hispanic white, black, Hispanic-Mexican, or other). Model 2, additionally adjusted for education level (less than high school, high school graduate, more than high school), smoking status (never, former, current), meeting recommended volume of physical activity (no/yes), alcohol consumption (male ≥ 20g/day, and female ≥ 10g/day), body mass index (kg/m2, continuous), systolic blood pressure (mmHg, continuous), the ratio of high-density lipoprotein to total cholesterol (ratio, continuous), type 2 diabetes (no/yes), stroked (no/yes), estimated glomerular filtration rate (≥ 60mL/min/1.73m², and <60 mL/min/1.73m²). Model 3, additionally adjusted for serum vitamin B12 (pmol/L, continuous). *P < 0.05, **P < 0.001. (DOCX) [file pone.0332987.s008.docx]

**Table S7. The B12 supplements subgroup analysis for the Relationship between Methylmalonic acid and Cognitions in NHANES 2011-2014**

|  | **Circulating methylmalonic acid (nmol/L)** | | | |  |
| --- | --- | --- | --- | --- | --- |
|  | **Q1 OR (95%CI)** | **Q2 OR (95%CI)** | **Q3 OR (95%CI)** | **Q4 OR (95%CI)** |  |
|  |  |  |  |  |  |
| **B12 supplements, yes** |  |  |  |  |  |
| DSST scores |  |  |  |  |  |
| Crude | 1.00(Ref.) | 0.74 (0.46 to 1.21) | 1.28 (0.76 to 2.17) | 2.17 (1.42 to 3.32)^**^ |  |
| Model 1 | 1.00(Ref.) | 0.63 (0.36 to 1.11) | 0.97 (0.53 to 1.77) | 1.75 (0.89 to 3.41) |  |
| Model 2 | 1.00(Ref.) | 0.48 (0.27 to 0.86) | 0.68 (0.36 to 1.30) | 1.23 (0.53 to 2.88) |  |
| Model 3 | 1.00(Ref.) | 0.54 (0.29 to 0.98)^*^ | 0.82 (0.43 to 1.57) | 1.51 (0.61 to 3.75) |  |
| AFT |  |  |  |  |  |
| Crude | 1.00(Ref.) | 0.87 (0.47 to 1.60) | 1.50 (0.90 to 2.50) | 1.61 (0.90 to 2.90) |  |
| Model 1 | 1.00(Ref.) | 0.78 (0.41 to 1.49) | 1.18 (0.64 to 2.19) | 1.24 (0.63 to 2.46) |  |
| Model 2 | 1.00(Ref.) | 0.72 (0.37 to 1.38) | 0.89 (0.43 to 1.81) | 0.85 (0.37 to 1.95) |  |
| Model 3 | 1.00(Ref.) | 0.70 (0.37 to 1.33) | 0.87 (0.44 to 1.70) | 0.83 (0.38 to 1.81) |  |
| CERAD: score immediate recall |  |  |  |  |  |
| Crude | 1.00(Ref.) | 0.97 (0.54 to 1.74) | 1.79 (1.11 to 2.88)^**^ | 1.94 (1.31 to 2.88)^**^ |  |
| Model 1 | 1.00(Ref.) | 0.82 (0.44 to 1.51) | 1.22 (0.77 to 1.95) | 1.32 (0.83 to 2.11) |  |
| Model 2 | 1.00(Ref.) | 0.68 (0.37 to 1.25) | 0.90 (0.55 to 1.46) | 0.92 (0.58 to 1.44) |  |
| Model 3 | 1.00(Ref.) | 0.67 (0.36 to 1.25) | 0.89 (0.54 to 1.45) | 0.90 (0.57 to 1.44) |  |
| CERAD: score delayed recall |  |  |  |  |  |
| Crude | 1.00(Ref.) | 1.37 (0.66 to 2.82) | 2.78 (1.62 to 4.75)^**^ | 2.79 (1.72 to 4.53)^**^ |  |
| Model 1 | 1.00(Ref.) | 1.09 (0.49 to 2.41) | 1.72 (1.00 to 2.97) | 1.67 (0.98 to 2.86) |  |
| Model 2 | 1.00(Ref.) | 1.05 (0.48 to 2.27) | 1.50 (0.79 to 2.86) | 1.18 (0.63 to 2.21) |  |
| Model 3 | 1.00(Ref.) | 1.06 (0.48 to 2.37) | 1.48 (0.75 to 2.92) | 1.14 (0.61 to 2.12) |  |
| **B12 supplements, no** |  |  |  |  |  |
| Variables |  |  |  |  |  |
| DSST scores |  |  |  |  |  |
| Crude | 1.00(Ref.) | 0.86 (0.56 to 1.31) | 0.86 (0.57 to 1.29) | 1.81 (1.33 to 2.47)^**^ |  |
| Model 1 | 1.00(Ref.) | 0.84 (0.56 to 1.27) | 0.94 (0.61 to 1.43) | 1.75 (1.23 to 2.51)^**^ |  |
| Model 2 | 1.00(Ref.) | 0.94 (0.58 to 1.54) | 1.02 (0.65 to 1.62) | 1.43 (0.89 to 2.32) |  |
| Model 3 | 1.00(Ref.) | 0.94 (0.57 to 1.54) | 1.01 (0.64 to 1.62) | 1.40 (0.84 to 2.32) |  |
| AFT |  |  |  |  |  |
| Crude | 1.00(Ref.) | 1.09 (0.58 to 2.04) | 0.85 (0.51 to 1.41) | 1.80 (1.07 to 3.05)^*^ |  |
| Model 1 | 1.00(Ref.) | 1.11 (0.59 to 2.07) | 0.88 (0.52 to 1.48) | 1.63 (0.96 to 2.76) |  |
| Model 2 | 1.00(Ref.) | 1.07 (0.56 to 2.05) | 0.85 (0.49 to 1.46) | 1.26 (0.71 to 2.25) |  |
| Model 3 | 1.00(Ref.) | 1.10 (0.57 to 2.13) | 0.88 (0.51 to 1.52) | 1.37 (0.77 to 2.45) |  |
| CERAD: score immediate recall |  |  |  |  |  |
| Crude | 1.00(Ref.) | 1.26 (0.79 to 2.03) | 1.30 (0.85 to 1.97) | 2.31 (1.50 to 3.54)^**^ |  |
| Model 1 | 1.00(Ref.) | 1.18 (0.68 to 2.05) | 1.17 (0.75 to 1.82) | 1.75 (1.05 to 2.91) |  |
| Model 2 | 1.00(Ref.) | 1.31 (0.71 to 2.43) | 1.17 (0.70 to 1.95) | 1.57 (0.83 to 2.96) |  |
| Model 3 | 1.00(Ref.) | 1.34 (0.72 to 2.49) | 1.20 (0.71 to 2.03) | 1.72 (0.91 to 3.25) |  |
| CERAD: score delayed recall |  |  |  |  |  |
| Crude | 1.00(Ref.) | 1.19 (0.75 to 1.86) | 1.18 (0.76 to 1.84) | 1.95 (1.39 to 2.73)^**^ |  |
| Model 1 | 1.00(Ref.) | 1.08 (0.67 to 1.74) | 1.01 (0.62 to 1.63) | 1.40 (0.90 to 2.18) |  |
| Model 2 | 1.00(Ref.) | 1.22 (0.71 to 2.08) | 1.00 (0.56 to 1.77) | 1.40 (0.83 to 2.38) |  |
| Model 3 | 1.00(Ref.) | 1.22 (0.71 to 2.10) | 1.01 (0.57 to 1.80) | 1.46 (0.84 to 2.53) |  |

Calculated using binary logistic regression;

Ref, treating the bottom group (the lowest quartile of MMA) as the reference;

Abbreviations: CI, confidence interval; OR, odds ratio; DSST, Digit Symbol Substitution Test; AFT, Animal Fluency test; CERAD, Consortium to Establish a Registry for Alzheimer’s Disease;

Model 1, adjusted for age (years, continuous), sex (female or male), and race/ethnicity (non-Hispanic white, black, Hispanic-Mexican, or other).

Model 2, additionally adjusted for education level (less than high school, high school graduate, more than high school), smoking status (never, former, current), meeting recommended volume of physical activity (no/yes), alcohol consumption (male ≥20g/day, and female ≥10g/day), body mass index (kg/m2, continuous), systolic blood pressure (mmHg, continuous), the ratio of high-density lipoprotein to total cholesterol (ratio, continuous), type 2 diabetes (no/yes), stroked (no/yes), estimated glomerular filtration rate (≥ 60mL/min/1.73m², and <60 mL/min/1.73m²).

Model 3, additionally adjusted for serum vitamin B12 (pmol/L, continuous).

^*^*P* < 0.05, ^**^*P*<0.001
