# Supplementary material for: Mitochondrial dysfunction-related metabolite methylmalonic acid is associated with decreased cognitive performance
Source: PLoS One. 2025 Oct 17;20(10):e0332987. doi: 10.1371/journal.pone.0332987 (PMC12533889; doi:10.1371/journal.pone.0332987)
Supplement: S8 Table — Calculated using binary logistic regression; Ref, treating the bottom group (the lowest quartile of MMA) as the reference; Abbreviations: CI, confidence interval; OR, odds ratio; DSST, Digit Symbol Substitution Test; AFT, Animal Fluency test; CERAD, Consortium to Establish a Registry for Alzheimer’s Disease. Model 1, adjusted for age (years, continuous), sex (female or male), and race/ethnicity (non-Hispanic white, black, Hispanic-Mexican, or other). Model 2, additionally adjusted for education level (less than high school, high school graduate, more than high school), smoking status (never, former, current), meeting recommended volume of physical activity (no/yes), alcohol consumption (male ≥ 20g/day, and female ≥ 10g/day), body mass index (kg/m2, continuous), systolic blood pressure (mmHg, continuous), the ratio of high-density lipoprotein to total cholesterol (ratio, continuous), type 2 diabetes (no/yes), stroked (no/yes), estimated glomerular filtration rate (≥ 60mL/min/1.73m², and <60 mL/min/1.73m²). Model 3, additionally adjusted for serum vitamin B12 (pmol/L, continuous). *P < 0.05, **P < 0.001. (DOCX) [file pone.0332987.s009.docx]

**Table S8. The sex subgroup analysis for the Relationship between Methylmalonic acid and Cognitions in NHANES 2011-2014**

|  | **Circulating methylmalonic acid (nmol/L)** | | | |
| --- | --- | --- | --- | --- |
|  | **Q1 OR (95%CI)** | **Q2 OR (95%CI)** | **Q3 OR (95%CI)** | **Q4 OR (95%CI)** |
|  |  |  |  |  |
| **Male** |  |  |  |  |
| DSST scores |  |  |  |  |
| Crude | 1.00(Ref.) | 1.16 (0.75 to 1.79) | 1.16 (0.69 to 1.95) | 2.39 (1.55 to 3.69)^**^ |
| Model 1 | 1.00(Ref.) | 1.21 (0.76 to 1.94) | 1.36 (0.77 to 2.40) | 2.75 (1.59 to 4.76)^**^ |
| Model 2 | 1.00(Ref.) | 1.43 (0.80 to 2.59) | 1.33 (0.68 to 2.60) | 2.69 (1.48 to 4.90)^**^ |
| Model 3 | 1.00(Ref.) | 1.44 (0.81 to 2.58) | 1.35 (0.70 to 2.59) | 2.74 (1.56 to 4.81)^**^ |
| AFT |  |  |  |  |
| Crude | 1.00(Ref.) | 1.32 (0.67 to 2.60) | 1.12 (0.56 to 2.22) | 2.26 (1.21 to 4.21)^**^ |
| Model 1 | 1.00(Ref.) | 1.30 (0.65 to 2.60) | 1.14 (0.54 to 2.37) | 2.15 (1.08 to 4.30)^*^ |
| Model 2 | 1.00(Ref.) | 1.42 (0.67 to 3.00) | 0.98 (0.44 to 2.21) | 1.75 (0.79 to 3.91) |
| Model 3 | 1.00(Ref.) | 1.40 (0.65 to 2.97) | 0.96 (0.42 to 2.18) | 1.68 (0.75 to 3.80) |
| CERAD: score immediate recall |  |  |  |  |
| Crude | 1.00(Ref.) | 1.37 (0.83 to 2.28) | 1.54 (0.99 to 2.39) | 2.39 (1.77 to 3.23)^**^ |
| Model 1 | 1.00(Ref.) | 1.26 (0.75 to 2.09) | 1.39 (0.88 to 2.19) | 1.97 (1.41 to 2.76)^**^ |
| Model 2 | 1.00(Ref.) | 1.35 (0.80 to 2.29) | 1.25 (0.75 to 2.07) | 1.63 (1.00 to 2.65) |
| Model 3 | 1.00(Ref.) | 1.44 (0.84 to 2.49) | 1.37 (0.80 to 2.35) | 1.87 (1.15 to 3.04)^*^ |
| CERAD: score delayed recall |  |  |  |  |
| Crude | 1.00(Ref.) | 1.54 (0.89 to 2.68) | 1.94 (1.21 to 3.12)^*^ | 2.88 (2.13 to 3.89)^**^ |
| Model 1 | 1.00(Ref.) | 1.35 (0.76 to 2.40) | 1.64 (0.98 to 2.75) | 2.22 (1.59 to 3.10)^**^ |
| Model 2 | 1.00(Ref.) | 1.45 (0.78 to 2.72) | 1.55 (0.77 to 3.11) | 2.10 (1.27 to 3.49)^**^ |
| Model 3 | 1.00(Ref.) | 1.44 (0.75 to 2.75) | 1.53 (0.73 to 3.18) | 2.06 (1.19 to 3.58)^*^ |
| **Female** |  |  |  |  |
| Variables |  |  |  |  |
| DSST score |  |  |  |  |
| Crude | 1.00(Ref.) | 0.57 (0.3 to 1.08) | 0.98 (0.59 to 1.61) | 1.87 (1.24 to 2.82)^**^ |
| Model 1 | 1.00(Ref.) | 0.50 (0.25 to 1.02) | 0.81 (0.46 to 1.45) | 1.47 (0.85 to 2.53) |
| Model 2 | 1.00(Ref.) | 0.43 (0.21 to 0.87)^*^ | 0.67 (0.35 to 1.28) | 0.79 (0.41 to 1.55) |
| Model 3 | 1.00(Ref.) | 0.46 (0.22 to 0.96)^*^ | 0.76 (0.40 to 1.46) | 0.91 (0.42 to 1.98) |
| AFT |  |  |  |  |
| Crude | 1.00(Ref.) | 0.77 (0.46 to 1.28) | 1.12 (0.75 to 1.66) | 1.51 (1.02 to 2.22)^*^ |
| Model 1 | 1.00(Ref.) | 0.73 (0.41 to 1.29) | 0.95 (0.62 to 1.45) | 1.13 (0.74 to 1.74) |
| Model 2 | 1.00(Ref.) | 0.58 (0.34 to 0.98)* | 0.76 (0.48 to 1.20) | 0.67 (0.45 to 1.00) |
| Model 3 | 1.00(Ref.) | 0.58 (0.34 to 0.99) | 0.78 (0.49 to 1.26) | 0.70 (0.47 to 1.06) |
| CERAD: score immediate recall |  |  |  |  |
| Crude | 1.00(Ref.) | 0.83 (0.50 to 1.36) | 1.41 (0.87 to 2.29) | 1.97 (1.28 to 3.02)^**^ |
| Model 1 | 1.00(Ref.) | 0.75 (0.41 to 1.37) | 1.02 (0.63 to 1.65) | 1.22 (0.78 to 1.92) |
| Model 2 | 1.00(Ref.) | 0.64 (0.33 to 1.22) | 0.80 (0.47 to 1.36) | 0.79 (0.49 to 1.29) |
| Model 3 | 1.00(Ref.) | 0.63 (0.33 to 1.19) | 0.78 (0.46 to 1.34) | 0.78 (0.50 to 1.22) |
| CERAD: score delayed recall |  |  |  |  |
| Crude | 1.00(Ref.) | 1.01 (0.54 to 1.91) | 1.57 (0.95 to 2.61) | 1.90 (1.15 to 3.14)^*^ |
| Model 1 | 1.00(Ref.) | 0.89 (0.46 to 1.74) | 1.06 (0.63 to 1.81) | 1.07 (0.58 to 1.95) |
| Model 2 | 1.00(Ref.) | 0.85 (0.43 to 1.70) | 0.93 (0.49 to 1.79) | 0.81 (0.40 to 1.62) |
| Model 3 | 1.00(Ref.) | 0.87 (0.43 to 1.76) | 0.93 (0.48 to 1.79) | 0.78 (0.40 to 1.55) |

Calculated using binary logistic regression;

Ref, treating the bottom group (the lowest quartile of MMA) as the reference;

Abbreviations: CI, confidence interval; OR, odds ratio; DSST, Digit Symbol Substitution Test; AFT, Animal Fluency test; CERAD, Consortium to Establish a Registry for Alzheimer’s Disease;

Model 1, adjusted for age (years, continuous), sex (female or male), and race/ethnicity (non-Hispanic white, black, Hispanic-Mexican, or other).

Model 2, additionally adjusted for education level (less than high school, high school graduate, more than high school), smoking status (never, former, current), meeting recommended volume of physical activity (no/yes), alcohol consumption (male ≥20g/day, and female ≥10g/day), body mass index (kg/m2, continuous), systolic blood pressure (mmHg, continuous), the ratio of high-density lipoprotein to total cholesterol (ratio, continuous), type 2 diabetes (no/yes), stroked (no/yes), estimated glomerular filtration rate (≥ 60mL/min/1.73m², and <60 mL/min/1.73m²).

Model 3, additionally adjusted for serum vitamin B12 (pmol/L, continuous).

^*^*P* < 0.05, ^**^*P*<0.001
