# Supplementary material for: Mitochondrial dysfunction-related metabolite methylmalonic acid is associated with decreased cognitive performance
Source: PLoS One. 2025 Oct 17;20(10):e0332987. doi: 10.1371/journal.pone.0332987 (PMC12533889; doi:10.1371/journal.pone.0332987)
Supplement: S9 Table — Calculated using binary logistic regression; Ref, treating the bottom group (the lowest quartile of MMA) as the reference; Abbreviations: CI, confidence interval; OR, odds ratio; DSST, Digit Symbol Substitution Test; AFT, Animal Fluency test; CERAD, Consortium to Establish a Registry for Alzheimer’s Disease. Model 1, adjusted for age (years, continuous), sex (female or male), and race/ethnicity (non-Hispanic white, black, Hispanic-Mexican, or other). Model 2, additionally adjusted for education level (less than high school, high school graduate, more than high school), smoking status (never, former, current), meeting recommended volume of physical activity (no/yes), alcohol consumption (male ≥ 20g/day, and female ≥ 10g/day), body mass index (kg/m2, continuous), systolic blood pressure (mmHg, continuous), the ratio of high-density lipoprotein to total cholesterol (ratio, continuous), type 2 diabetes (no/yes), stroked (no/yes), estimated glomerular filtration rate (≥ 60mL/min/1.73m², and <60 mL/min/1.73m²). Model 3, additionally adjusted for serum vitamin B12 (pmol/L, continuous). *P < 0.05, **P < 0.001. (DOCX) [file pone.0332987.s010.docx]

**Table S9. The age-related subgroup analysis for the Relationship between Methylmalonic acid and Cognitions in NHANES 2011-2014**

|  | **Circulating methylmalonic acid (nmol/L)** | | | |
| --- | --- | --- | --- | --- |
|  | **Q1 OR (95%CI)** | **Q2 OR (95%CI)** | **Q3 OR (95%CI)** | **Q4 OR (95%CI)** |
|  |  |  |  |  |
| **age>75** |  |  |  |  |
| DSST scores |  |  |  |  |
| Crude | 1.00(Ref.) | 0.81 (0.53 to 1.23) | 0.81 (0.52 to 1.25) | 1.95 (1.31 to 2.90)^**^ |
| Model 1 | 1.00(Ref.) | 0.92 (0.59 to 1.43) | 1.03 (0.67 to 1.58) | 2.62 (1.76 to 3.91)^**^ |
| Model 2 | 1.00(Ref.) | 0.48 (0.25 to 0.91)* | 0.74 (0.31 to 1.78) | 0.95 (0.43 to 2.12) |
| Model 3 | 1.00(Ref.) | 0.89 (0.48 to 1.65) | 0.85 (0.54 to 1.34) | 1.57 (0.94 to 2.64) |
| AFT |  |  |  |  |
| Crude | 1.00(Ref.) | 1.03 (0.61 to 1.74) | 0.87 (0.50 to 1.54) | 1.56 (0.98 to 2.51) |
| Model 1 | 1.00(Ref.) | 1.09 (0.64 to 1.86) | 0.96 (0.55 to 1.70) | 1.64 (1.02 to 2.66)^*^ |
| Model 2 | 1.00(Ref.) | 0.63 (0.26 to 1.53) | 0.80 (0.39 to 1.61) | 0.84 (0.45 to 1.57) |
| Model 3 | 1.00(Ref.) | 0.97 (0.55 to 1.72) | 0.85 (0.48 to 1.48) | 1.18 (0.69 to 2.02) |
| CERAD: score immediate recall |  |  |  |  |
| Crude | 1.00(Ref.) | 1.10 (0.69 to 1.74) | 1.18 (0.76 to 1.83) | 1.88 (1.41 to 2.49)^**^ |
| Model 1 | 1.00(Ref.) | 1.09 (0.68 to 1.75) | 1.18 (0.80 to 1.74) | 1.82 (1.38 to 2.39)^**^ |
| Model 2 | 1.00(Ref.) | 0.66 (0.36 to 1.20) | 0.80 (0.34 to 1.89) | 0.85 (0.41 to 1.76) |
| Model 3 | 1.00(Ref.) | 1.13 (0.66 to 1.91) | 1.10 (0.74 to 1.65) | 1.49 (1.01 to 2.18)^*^ |
| CERAD: score delayed recall |  |  |  |  |
| Crude | 1.00(Ref.) | 1.42 (0.84 to 2.39) | 1.37 (0.89 to 2.11) | 1.84 (1.29 to 2.62)^**^ |
| Model 1 | 1.00(Ref.) | 1.38 (0.82 to 2.34) | 1.33 (0.88 to 2.00) | 1.69 (1.17 to 2.46)^*^ |
| Model 2 | 1.00(Ref.) | 0.61 (0.27 to 1.38) | 1.01 (0.53 to 1.95) | 1.01 (0.54 to 1.90) |
| Model 3 | 1.00(Ref.) | 1.41 (0.82 to 2.44) | 1.19 (0.70 to 2.02) | 1.40 (0.86 to 2.29) |
| **Age≤75** |  |  |  |  |
| DSST scores |  |  |  |  |
| Crude | 1.00(Ref.) | 0.49 (0.28 to 0.86)^*^ | 0.65 (0.36 to 1.16) | 0.89 (0.53 to 1.49) |
| Model 1 | 1.00(Ref.) | 0.49 (0.26 to 0.93)^*^ | 0.77 (0.37 to 1.61) | 1.13 (0.58 to 2.19) |
| Model 2 | 1.00(Ref.) | 0.84 (0.47 to 1.51) | 0.83 (0.50 to 1.36) | 1.55 (0.93 to 2.60) |
| Model 3 | 1.00(Ref.) | 0.54 (0.27 to 1.11) | 0.93 (0.39 to 2.15) | 1.21 (0.50 to 2.90) |
| AFT |  |  |  |  |
| Crude | 1.00(Ref.) | 0.60 (0.28 to 1.27) | 0.88 (0.51 to 1.53) | 1.08 (0.61 to 1.91) |
| Model 1 | 1.00(Ref.) | 0.62 (0.28 to 1.39) | 0.98 (0.53 to 1.82) | 1.22 (0.66 to 2.25) |
| Model 2 | 1.00(Ref.) | 0.96 (0.54 to 1.71) | 0.82 (0.46 to 1.45) | 1.31 (0.78 to 2.21) |
| Model 3 | 1.00(Ref.) | 0.61 (0.25 to 1.50) | 0.84 (0.40 to 1.73) | 0.90 (0.49 to 1.66) |
| CERAD: score immediate recall |  |  |  |  |
| Crude | 1.00(Ref.) | 0.72 (0.39 to 1.33) | 1.00 (0.50 to 1.99) | 1.06 (0.55 to 2.02) |
| Model 1 | 1.00(Ref.) | 0.72 (0.39 to 1.34) | 1.05 (0.49 to 2.28) | 1.11 (0.54 to 2.27) |
| Model 2 | 1.00(Ref.) | 1.11 (0.67 to 1.84) | 1.08 (0.74 to 1.59) | 1.47 (1.03 to 2.11)* |
| Model 3 | 1.00(Ref.) | 0.63 (0.35 to 1.13) | 0.74 (0.31 to 1.80) | 0.77 (0.37 to 1.58) |
| CERAD: score delayed recall |  |  |  |  |
| Crude | 1.00(Ref.) | 0.62 (0.29 to 1.35) | 1.19 (0.63 to 2.26) | 1.22 (0.66 to 2.26) |
| Model 1 | 1.00(Ref.) | 0.59 (0.26 to 1.33) | 1.10 (0.57 to 2.11) | 1.12 (0.59 to 2.13) |
| Model 2 | 1.00(Ref.) | 1.44 (0.83 to 2.49) | 1.31 (0.79 to 2.18) | 1.59 (1.01 to 2.52) |
| Model 3 | 1.00(Ref.) | 0.65 (0.29 to 1.46) | 1.06 (0.53 to 2.10) | 1.03 (0.53 to 2.01) |

Calculated using binary logistic regression;

Ref, treating the bottom group (the lowest quartile of MMA) as the reference;

Abbreviations: CI, confidence interval; OR, odds ratio; DSST, Digit Symbol Substitution Test; AFT, Animal Fluency test; CERAD, Consortium to Establish a Registry for Alzheimer’s Disease;

Model 1, adjusted for age (years, continuous), sex (female or male), and race/ethnicity (non-Hispanic white, black, Hispanic-Mexican, or other).

Model 2, additionally adjusted for education level (less than high school, high school graduate, more than high school), smoking status (never, former, current), meeting recommended volume of physical activity (no/yes), alcohol consumption (male ≥20g/day, and female ≥10g/day), body mass index (kg/m2, continuous), systolic blood pressure (mmHg, continuous), the ratio of high-density lipoprotein to total cholesterol (ratio, continuous), type 2 diabetes (no/yes), stroked (no/yes), estimated glomerular filtration rate (≥ 60mL/min/1.73m², and <60 mL/min/1.73m²).

Model 3, additionally adjusted for serum vitamin B12 (pmol/L, continuous).

^*^*P* < 0.05, ^**^*P*<0.001
